# A positive feedback loop between SMAD3 and PINK1 in regulation of mitophagy

**DOI:** 10.1038/s41421-025-00774-4

**Published:** 2025-03-11

**Authors:** Mingzhu Tang, Dade Rong, Xiangzheng Gao, Guang Lu, Haimei Tang, Peng Wang, Ning-Yi Shao, Dajing Xia, Xin-Hua Feng, Wei-Feng He, Weilin Chen, Jia-Hong Lu, Wei Liu, Han-Ming Shen

**Affiliations:** 1 Faculty of Healthy Sciences, Ministry of Education Frontiers Science Center for Precision Oncology, University of Macau, Macau, China; 2 Zhongshan School of Medicine, Sun Yat-sen University, Guangzhou, Guangdong China; 3 Department of Immunology, Shenzhen University School of Medicine, Shenzhen, Guangdong China; 4 Department of Toxicology of School of Public Health and Department of Gynecologic Oncology of Women’s Hospital, Zhejiang University School of Medicine, Hangzhou, Zhejiang China; 5 Life Science Institute, Zhejiang University, Hangzhou, Zhejiang China; 6 State Key Laboratory of Trauma, Burn and Combined Injury, Institute of Burn Research, Southwest Hospital, Army Medical University, Chongqing, China; 7 State Key Laboratory of Quality Research in Chinese Medicine, Institute of Chinese Medical Sciences, University of Macau, Macau, China; 8 Center for Metabolism Research, the Fourth Affiliated Hospital of Zhejiang University School of Medicine, and International School of Medicine, International Institutes of Medicine, Zhejiang University, Yiwu, Zhejiang China

**Keywords:** Mitophagy, Phosphorylation

## Abstract

PTEN-induced kinase-1 (PINK1) is a crucial player in selective clearance of damaged mitochondria via the autophagy-lysosome pathway, a process termed mitophagy. Previous studies on PINK1 mainly focused on its post-translational modifications, while the transcriptional regulation of PINK1 is much less understood. Herein, we reported a novel mechanism in control of *PINK1* transcription by SMAD Family Member 3 (SMAD3), an essential component of the transforming growth factor beta (TGFβ)-SMAD signaling pathway. First, we observed that mitochondrial depolarization promotes *PINK1* transcription, and SMAD3 is likely to be the nuclear transcription factor mediating *PINK1* transcription. Intriguingly, SMAD3 positively transactivates *PINK1* transcription independent of the canonical TGFβ signaling components, such as TGFβ-R1, SMAD2 or SMAD4. Second, we found that mitochondrial depolarization activates SMAD3 via PINK1-mediated phosphorylation of SMAD3 at serine 423/425. Therefore, PINK1 and SMAD3 constitute a positive feedforward loop in control of mitophagy. Finally, activation of *PINK1* transcription by SMAD3 provides an important pro-survival signal, as depletion of SMAD3 sensitizes cells to cell death caused by mitochondrial stress. In summary, our findings identify a non-canonical function of SMAD3 as a nuclear transcriptional factor in regulation of *PINK1* transcription and mitophagy and a positive feedback loop via PINK1-mediated SMAD3 phosphorylation and activation. Understanding this novel regulatory mechanism provides a deeper insight into the pathological function of PINK1 in the pathogenesis of neurodegenerative diseases such as Parkinson's disease.

## Introduction

To maintain mitochondrial quality control and cellular homeostasis, dysfunctional mitochondria are selectively degraded through the autophagy-lysosome pathway, a process termed as mitophagy^1^. Defects in mitophagy has been implicated in various human disorders such as cancer, neurodegenerative and cardiovascular diseases^2,3^. Multiple molecular mechanisms of mitophagy have been reported, and among them the mechanism involving Phosphatase and Tensin homolog (PTEN)-induced kinase 1 (PINK1) and the E3 ubiquitin (Ub) ligase Parkin has been extensively studied as a critical player in control of mitophagy in response to mitochondrial stress^4,5^.

PINK1, a serine/threonine kinase, once synthesized in the cytosol, is readily imported into mitochondria via the translocases of the outer membrane (TOM) complex, where it is subjected to cleavage and degradation by mitochondrial proteases presenilin-associated rhomboid-like protein (PARL) and mitochondrial processing peptidase^6–8^. The remaining C-terminal of PINK1 is then retrieved back to the cytosol and quickly degraded by the proteasome via the N-end rule pathway^9^. Upon mitochondrial damage or depolarization, the above-described process of PINK1 proteolysis is inhibited, leading to its accumulation and stabilization on the outer mitochondrial membrane (OMM)^8,10^. The stabilized PINK1 forms a homodimer, leading to its autophosphorylation and activation. The activated PINK1 then directly phosphorylates Ub^11–13^ and Parkin, both at Ser65^14–16^. Notably, the phosphorylated Ub chains also function as a Parkin activator, which in turn, activates Parkin and recruits more Ub at the OMM for PINK1 phosphorylation^17–20^. Therefore, PINK1-mediated phosphorylation and Parkin-mediated ubiquitination form a positive feedforward loop for the induction of mitophagy. All these findings highlight a fundamental role of PINK1 in regulating mitophagy through its post-translational modifications (PTMs).

Unwarranted and dysregulated PINK1 is harmful to cell survival and needs to be tightly regulated^21^. In contrast to the well-studied PTMs of PINK1, at present, its transcriptional modulation under mitochondrial stress remains largely unexplored. At present, several nuclear transcriptional factors have been reported to regulate *PINK1* transcription, such as nuclear forehead box O3 (FoxO3) under oxidative stress condition^22^. In addition, some other nuclear proteins such as lysine acetyltransferase 8 (KAT8) and KAT8 Regulatory NSL Complex Subunit 1 (KANSL1) have also been reported to regulate PINK1 function^23^. However, in the context of mitophagy induced by acute mitochondrial damage, the transcriptional regulation of PINK1 has not been fully investigated.

In this study, by combining bio-informatic analysis and biochemical as well as cell biology approaches, we disclose a novel function of SMAD3 in regulation of *PINK1* transcription and mitophagy, a process independent of the canonical TGFβ signaling pathway. Importantly, SMAD3 is directly phosphorylated and activated by PINK1, thereby constituting a positive feedforward loop between SMAD3 and PINK1 for induction of mitophagy. Our findings thus shed new lights on the molecular mechanisms of mitophagy via regulation of *PINK1* transcription. This study might provide rationales for development of interventional strategies for mitophagy-related disorders such as Parkinson's disease (PD) by targeting the SMAD3-PINK1 signaling loop.

## Results

### Association of SMAD3 with PINK1 expression

**Fig. 1:**
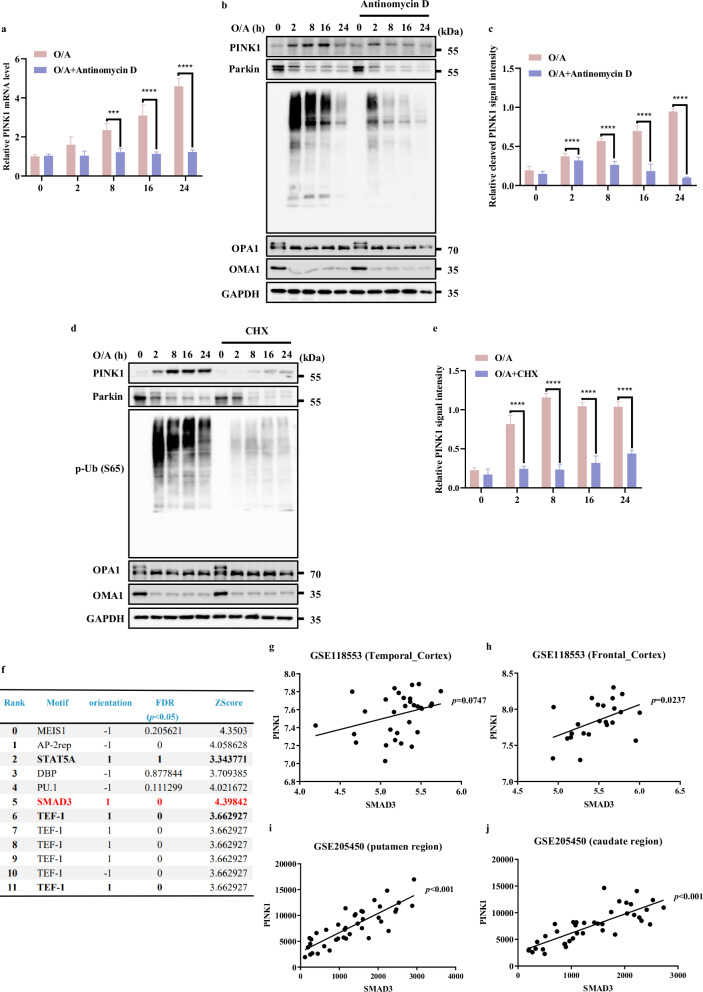
Association of SMAD3 with PINK1 expression. **a** *PINK1* mRNA level was quantified using RT-PCR in HeLa YFP-Parkin cells followed by with or without O/A (1/1 μM) induction for the indicated time in the presence or absence of Antinomycin D (2 μg/mL) pre-treatment. **b**, **d** HeLa YFP-Parkin cells were pre-treated with or without Antinomycin D (2 μg/mL) (**b**) or CHX (50 μM) (**c**) for 1 h. Subsequently, the cells were treated with O/A (1/1 μM) for the indicated time and subjected to western blotting analysis with the indicated antibodies. **c** The indicated proteins were quantified by normalizing to GAPDH as loading control in cells treated as in **b**. **e** The indicated proteins were quantified by normalizing to GAPDH as loading control in cells treated as in **d**. **f** Bioinformatics analysis about the prediction of transcriptional factors for *PINK1* by (MotifMap | RNA (uci.edu). Red labeling indicates the most possible transcription factor for *PINK1* among the top candidates. **g**, **h** The correlation analysis between PINK1 and SMAD3 in human temporal cortex, and frontal cortex from cohort-118553 (GEO database). **i**, **j** The correlation analysis between PINK1 and SMAD3 in human putamen and caudate region from cohort-GSE205450 (GEO database). Data shown represent the means ± SD of three biological replicates, ***P < 0.001, ****P < 0.0001; significance was determined by one-way ANOVA test followed by Tukey’s correction (**a**, **c**, **e**).

To understand the regulatory mechanisms of PINK1 at the transcriptional level, we first treated HeLa cells stably expressing YFP-Parkin (HeLa YFP-Parkin cells) with two mitochondrial damage agents oligomycin and antimycin A (O/A) to induce PINK1 stabilization on the depolarized mitochondria^24^. An upregulation of mRNA and protein level of PINK1 were detected (Fig. 1a‒c). Of note, treatment with Actinomycin D, an inhibitor of de novo mRNA synthesis, almost completely abolished both the mRNA and protein level of PINK1 (Fig. 1a‒c), suggesting that PINK1 is under active transcription upon mitochondrial damage. Consistently, there is also a remarkable reduction of PINK1 protein level under the treatment of cycloheximide (CHX) to block de novo protein synthesis (Fig. 1d, e).

Since the loss of MMP is considered as a driver to promote PINK1 accumulation^16^, we wondered whether the reduction of full length PINK1 caused by CHX and Actinomycin D was due to the recovery of MMP. To test this possibility, we used TMRE staining to detect MMP^25^. As shown in Supplementary Fig. S1a, O/A treatment resulted in a significant decrease in MMP, which was not affected by the pre-treatment with CHX and Actinomycin D. It is well-known that O/A-induced the activation and degradation of Zinc Metallopeptidase (OMA1) can cleave long-active L-OPA1 (Mitochondrial Dynamin Like GTPase) to fusion-inactive S-OPA1 isoforms^26^. As shown in Figs. 1b, d, OMA1 and its well-known substrate OPA1 were also not affected by CHX and Actinomycin D, suggesting that inhibition of mRNA and protein synthesis does not affect mitochondrial depolarization.

To rule out the possibility that the decreased level of PINK1 in response to Actinomycin D and CHX was due to increased cell death, cell viability was measured by propidium iodide (PI) exclusion assay coupled with flow cytometry. The results showed that the presence of Actinomycin D and CHX did not change the cell viability under treatment with O/A for 24 h (Supplementary Fig. S1b). Thus, it is believed that the reduction of *PINK1* mRNA and protein level caused by Actinomycin D and CHX are not caused by increased cell death.

In addition, we were able to observe similar trends of changes of PINK1 mRNA and protein levels in HeLa cells without Parkin expression (Supplementary Fig. S1c‒e) and HEK293T cells with endogenously expressed Parkin (Supplementary Fig. S1g‒i), which also showed no effect of pre-treatment of Actinomycin D and CHX on O/A-induced decrease of MMP (Supplementary Fig. S1f, j).

Here we also used a mitochondrial uncoupler carbonyl cyanide chlorophenylhydrazone (CCCP) and observed similar effects on *PINK1* mRNA level as O/A (Supplementary Fig. S1m). Moreover, we quantified *PINK1* mRNA level by using two additional primers targeting different regions of *PINK1* cDNA (Supplementary Fig. S1k) and the specific size of qPCR product by these three primers were shown in Supplementary Fig. S1l. Notably, there was a similar trend in cells treated with CCCP with or without Actinomycin D (Supplementary Fig. S1m). All these data suggest that PINK1 is under active transcription and translation upon mitochondrial damage, a process independent of Parkin.

To identify the potential transcriptional factors in control of *PINK1* transcription under mitochondrial damage, we conducted a bioinformatics prediction analysis (MotifMap | RNA (uci.edu), https://motifmap-rna.ics.uci.edu/search)^27^ and several potential candidates were identified, of which we focused on SMAD Family Member 3 (SMAD3) based on its positive orientation, statistical significance and score ranking (Fig. 1f). Furthermore, correlation analysis using a human brain cohort-GSE118553 (acquired from GEO database) revealed a positive correlation between the mRNA levels of *PINK1* and *SMAD3*, but not *SMAD2* and *SMAD4*, in temporal cortex and frontal cortex (Fig. 1g, h; Supplementary S1n‒q). In line with this, the same result could also be obtained from another independent human brain cohort-GSE205450, in which positive correlative relationships between *PINK1* and *SMAD3* in putamen and caudate region were found (Fig. 1i, j).

Consistently, SMAD3 is also listed as one of the positive modulators of Parkin translocation in selective mitophagy according to the Genome-wide high-content siRNA screen^28^, which further supports the relevance between SMAD3 and PINK1. Taken together, these results suggest that PINK1 is transcriptionally upregulated under mitochondrial stress whereas SMAD3 is possibly implicated.

### SMAD3 is a positive nuclear transcription factor of PINK1

**Fig. 2:**
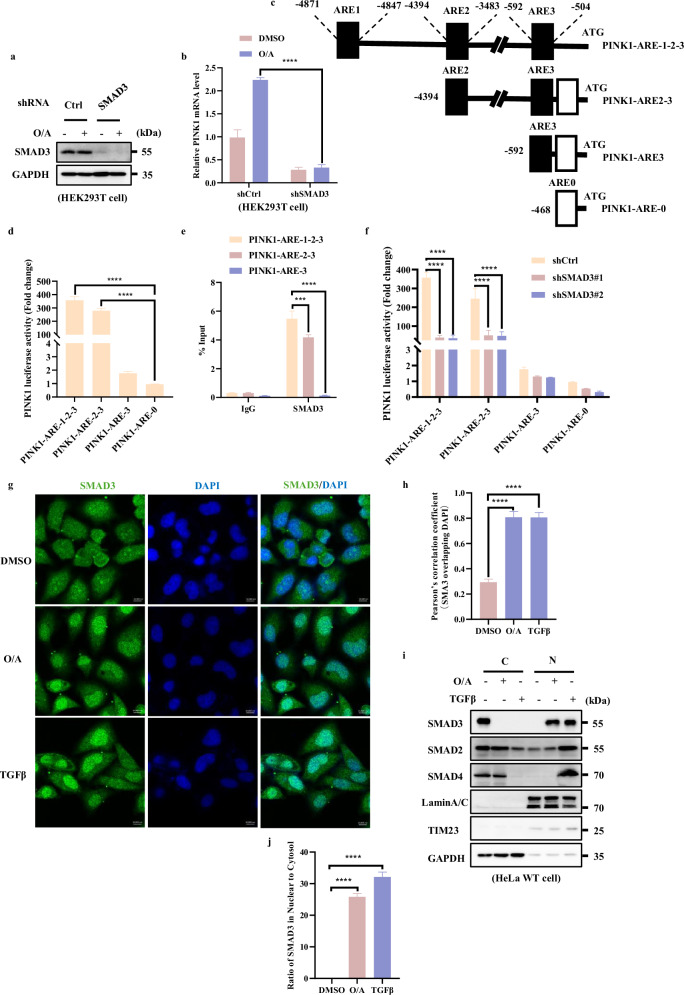
SMAD3 is a positive nuclear transcription factor of PINK1. **a** HEK293T WT and SMAD3 knockdown cells were treated with O/A (1/1 μM) for 4 h and harvested for western blotting analysis with the indicated antibodies. **b** *PINK1* mRNA level was quantified using RT-PCR in HEK293T SMAD3 knockdown cells followed with or without O/A (1/1 μM) induction for 4 h. **c** Schematic presentation of SMAD3 and *PINK1* binding sites. The scheme represents the *PINK1* promoter construct lacking different SMAD3 response elements (ARE1, ARE2, ARE3). PINK1-ARE-0 is shown as a negative control. **d** Different *PINK1* promoter constructs (PINK1-ARE-1-2-3, PINK1-ARE-2-3, PINK1-ARE-3) were co-transfected in HEK293T cells with the *pRL-TK* reporter gene (normalize transfection efficiencies) for 24 h. Data are expressed as a percentage of firefly luciferase activity (promoter constructs-transfected cells)/Renilla luciferase activity (pRL-TK-transfected cells) by Dual-Glo luciferase assay system and presented as the means ± SD of 3 independent experiments performed in triplicates. **e** ChIP analysis of SMAD3 binding to the *PINK1* locus in HEK293T cells. *GAPDH* was used as a negative control. **f** Different *PINK1* promoter construct (PINK1-ARE-1-2-3, PINK1-ARE-2-3, PINK1-ARE-3) were co-transfected in HEK293T cells or HEK293T SMAD3 knockdown cell with the *pRL-TK* reporter gene (normalize transfection efficiencies) for 24 h. Data are expressed as a percentage of firefly luciferase activity (promoter constructs-transfected cells)/Renilla luciferase activity (pRL-TK-transfected cells) by Dual-Glo luciferase assay system and presented as the means ± SD of 3 independent experiments performed in triplicates. **g** HeLa WT cells were treated with OA (1/1 μM) for 4 h and analyzed by confocal microscopy followed by immune-staining with SMAD3 (red) and DAPI. Scale bars, 10 μm. **h** The colocalization of SMAD3 with DAPI in **g** was quantified by Pearson’s correlation overlapping coefficient. **i** Subcellular fractionation was performed to isolate the cytosolic and nuclear fractions in HeLa WT cells treated with O/A (1 μM) for 4 h. The samples were then analyzed by western blotting with the indicated antibodies. Lamin A/C and GAPDH were used as nuclear and cytosolic markers, respectively. **j** SMAD3 in nucleus was quantified by normalizing to that in cytosol of cells treated as in **i**. Data shown represent the means ± SD of three biological replicates, ***P < 0.001, ****P < 0.0001; significance was determined by one-way ANOVA test followed by Tukey’s correction (**b**, **d**, **e**, **f**, **h**, **j**).

Since SMAD3 is a well-known transcription factor^29^, the positive association between *PINK1* mRNA levels and SMAD3 shown above indicates the possibility that SMAD3 is the potential transcription factor for PINK1. To test this hypothesis, we first tested the cells with stable knockdown of SMAD3 and found that SMAD3 deficiency resulted in a significant downregulation of *PINK1* mRNA level induced by O/A (Fig. 2a, b; Supplementary S2a‒f), indicating that SMAD3 plays an important role in mediating *PINK1* transcription. Next, we performed a chromatin immunoprecipitation (ChIP)-seq assay through immunoprecipitating DNA with SMAD3 antibody and identified three potential putative SMAD3 responsive elements in the *PINK1* promoter region (ARE1, ‒4871 ~ ‒4847 bp; ARE2, ‒4394 ~ ‒3483 bp; ARE3, ‒592 ~ ‒504 bp) (Fig. 2c). We then generated different mutations by region-directed deletion of these AREs (Fig. 2c). The luciferase reporter assay revealed that cells with PINK1-ARE-0 (with deletion of ARE1, ARE2 and ARE3) and PINK1-ARE-3 (with deletion of ARE1 and ARE2) dramatically decreased the reporter activity, suggesting that the two responsive elements (ARE1, ‒4871 ~ ‒4847 bp; ARE2, ‒4394 ~ ‒3483 bp) are required for SMAD3-mediatd *PINK1* transcription (Fig. 2d). This result was further supported by the ChIP assay, in which deletion of ARE1 and ARE2 let to remarked reduction of SMAD3 recruitment to the *PINK1* promoter region (Fig. 2e). Furthermore, knockdown of SMAD3 significantly reduced the PINK1 luciferase activity only in cells with expression of PINK1-ARE-1-2-3 and PINK1-ARE-2-3, but not in cells with expression of PINK1-ARE-3 and PINK1-ARE-0 (Fig. 2f). Together, the above data suggest that the transcriptional regulation of *PINK1* by SMAD3 is mainly dependent on the ARE1 and ARE2 elements.

It is well-known that in response to transforming growth factor beta (TGFβ), SMAD3 is phosphorylated at Ser423/425, which is necessary for its nuclear translocation to drive its target gene transcription^30–32^. Thus, we examined the cellular localization of SMAD3 under mitochondrial damaging condition and found that, similar to the results obtained with TGFβ-treated cells, there is a drastically enhanced SMAD3 nuclear translocation after O/A treatment (Fig. 2g, h). In agreement with the results obtained by fluorescence assay, O/A treatment led to an accumulation of SMAD3 in the nuclear fraction (Fig. 2i, j). These data collectively suggest that SMAD3 is capable of promoting *PINK1* transcription upon mitochondrial damage.

### SMAD3 up-regulates PINK1 expression

**Fig. 3:**
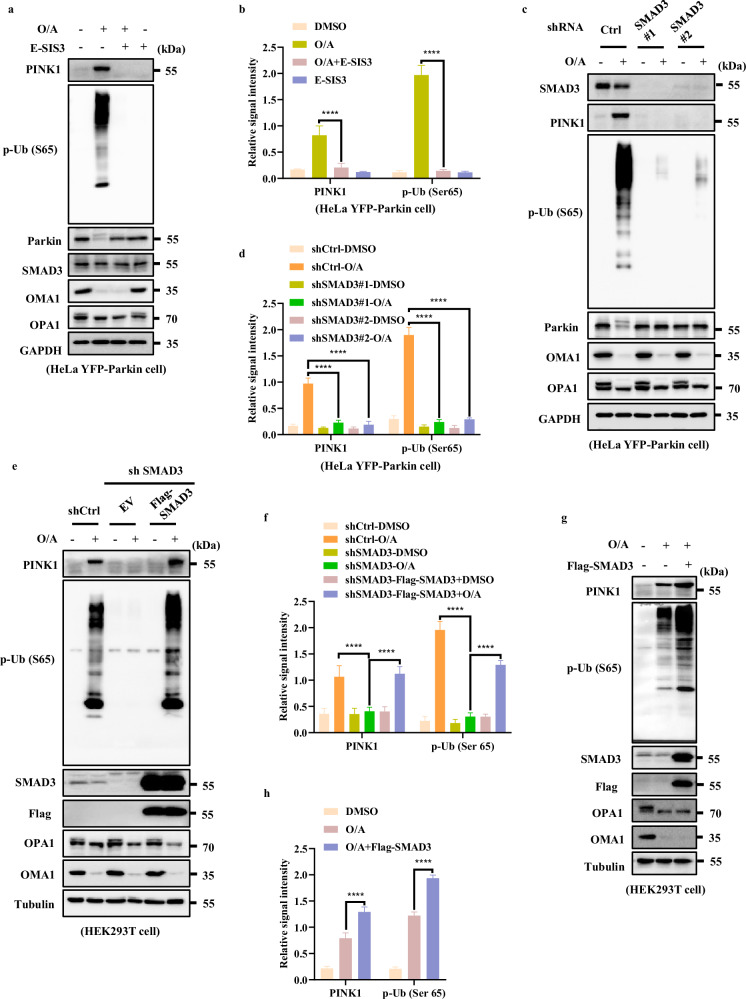
SMAD3 up-regulates PINK1 expression. **a** HeLa YFP-Parkin cells were pre-treated with E-SIS3 (20 μM) for 1 h. Subsequently, the cells were treated with or without O/A (1/1 μM) for 4 h and subjected to western blotting analysis with the indicated antibodies. **b** The indicated proteins were quantified by normalizing to GAPDH as loading control in cells treated as in **a**. **c** SMAD3 level was detected by infection with control, shSMAD3#1 or shSMAD3#2 lentiviral particles in HeLa YFP-Parkin cells. Cells were then incubated with OA (1/1 μM) for 4 h for western blotting analysis with the indicated antibodies. **d** The indicated proteins were quantified by normalizing to GAPDH as loading control in cells treated as in **c**. **e** HEK293T WT and SMAD3-knockdown cells transfected with either empty vector (EV) or Flag-SMAD3 for 24 h were treated with O/A (1/1 μM) and harvested at the indicated time points for western blotting analysis with the indicated antibodies. **f** The indicated proteins were quantified by normalizing to α-Tubulin as loading control in cells treated as in **e**. **g** HEK293T cells transfected with Flag-SMAD3 for 24 h were treated with O/A (1/1 μM) and harvested at the indicated time points for western blotting analysis with the indicated antibodies. **h** The indicated proteins were quantified by normalizing to α-Tubulin as loading control in cells treated as in **g**. Data shown represent the means ± SD of three biological replicates, ****P < 0.0001; significance was determined by one-way ANOVA test followed by Tukey’s correction (**b**, **d**, **f**, **h**).

Next, we examined the impact of SMAD3 on PINK1 expression by both pharmacological and genetic approaches. First, we blocked SMAD3 by E-SIS3, a potent and specific inhibitor of SMAD3 known to suppress SMAD3 phosphorylation and hence its transcriptional function^33,34^. As shown in Fig. 3a, b, the accumulation of full-length PINK1 and its downstream p-Ub (Ser65) levels were markedly reduced in the presence of E-SIS3 in cells treated with O/A.

It has been shown that inhibition of SMAD3 via E-SIS3 is linked to decreased cell viability^35^. To exclude the involvement of cell death in the reduction of PINK1 protein level, cell viability was measured and the results showed that the presence or absence of E-SIS3 had no impact on cell viability (Supplementary Fig. S3a). Besides, we also showed that the inhibition of SMAD3 has no effect on O/A-induced loss of MMP as well as the cleavage of OMA1 and OPA1 (Fig. 3a; Supplementary Fig. S3b), indicating that SMAD3 suppression does not affect mitochondrial depolarization.

Moreover, we generated SMAD3 stable knockdown cells and found that shSMAD3 resulted in a significant decrease of full-length PINK1 and p-Ub (Ser65) in cells treated with O/A (Fig. 3c, d). Similar changes were observed in HeLa cells without expression of Parkin (Supplementary Fig. S3d, g, h, k), HEK293T cells (Supplementary Fig. S3e, i, l) and SY5Y cells (Supplementary Fig. S3f, j) with endogenous expression of Parkin, suggesting that the regulatory effect of SMAD3 on PINK1 is independent of Parkin. Similarly, deletion of SMAD3 did not reverse MMP in response to O/A treatment (Supplementary Fig. S3c), thus confirming that MMP is not implicated in the reduction of PINK1 protein level caused by SMAD3 suppression.

To further confirm the role of SMAD3 in regulation of PINK1 expression, we performed reconstitution of Flag-SMAD3 in cells with stable knockdown of SMAD3 and observed that the reconstituted SMAD3 was able to restore PINK1 and p-Ub (Ser65) level in SMAD3-knockdown cells treated with O/A (Fig. 3e, f). Moreover, SMAD3 overexpression also led to significant increase of PINK1 protein level (Fig. 3g, h). Therefore, we conclude here that SMAD3 is capable of upregulating PINK1 expression upon mitochondrial damage.

### SMAD3 promotes mitophagy

**Fig. 4:**
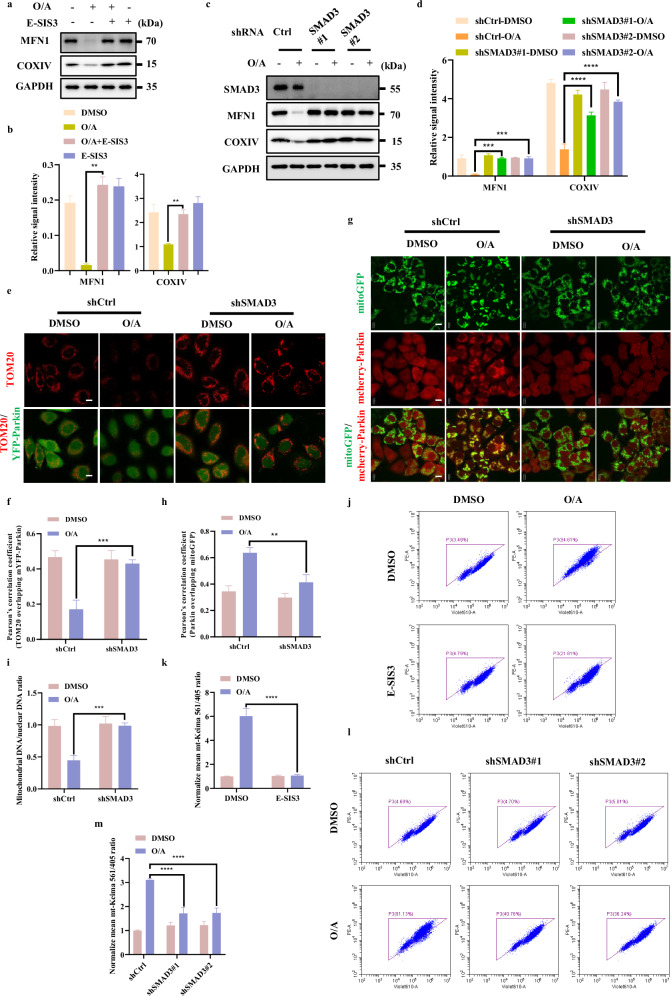
SMAD3 promotes mitophagy. **a** HeLa YFP-Parkin cells were pre-treated with E-SIS3 (20 μM) for 1 h. Subsequently, the cells were treated with or without OA (1/1 μM) for 4 h and subjected to western blotting analysis with the indicated antibodies. **b** The indicated proteins were quantified by normalizing to GAPDH as loading control in cells treated as in **a**. **c** SMAD3 level was detected by infection with control, shSMAD3#1 or shSMAD3#2 lentiviral particles in HeLa YFP-Parkin cells. Cells were then incubated with OA (1/1 μM) for 4 h for western blotting analysis with the indicated antibodies. **d** The indicated proteins were quantified by normalizing to GAPDH as loading control in cells treated as in **c**. **e** HeLa YFP-Parkin WT and SMAD3-knockdown cells were treated with OA (1/1 μM) for 24 h and analyzed by confocal microscopy followed by immuostained with an anti-TOMM20 antibody (Red) and DAPI. Scale bars, 10 µm. **f** The colocalization of TOM20 with YFP-Parkin in **e** was quantified by Pearson’s correlation overlapping coefficient. **g** HeLa WT cells with stable expression of mcherry-Parkin (Red) and mito-GFP (Green) were infected with shcontrol or shSMAD3 lentiviral particles specific shRNA. HeLa mcherry-Parkin and SMAD3-knockdown cells were treated with OA (1/1 μM) for 60 min and analyzed for mcherry-Parkin (Red) and mito-GFP (Green) by confocal microscopy. Scale bars, 10 µm. **h** The colocalization of mcherry-Parkin with mito-GFP in **g** was quantified by Pearson’s correlation overlapping coefficient. **i** Quantification of mtDNA by qPCR through amplifying mtDNA gene *ND1* after treatment with O/A (1/1 µM) for 24 h. **j** HeLa YFP-Parkin mito-Keima cells were pre-treated with E-SIS3 (20 μM) for 1 h and treated with OA (1/1 μM) for 8 h. Subsequently, the cells were harvested for FACS analysis by excitation at 405 nm (neutral pH) and 561 nm (acidic pH). **k** The relative mean mt-Keima 561/405 ratio was quantified by normalizing to DMSO group in cells treated as in **j**. **l** HeLa YFP-Parkin mito-Keima WT and SMAD3-knockdown cells were treated with OA (1/1 μM) for 8 h and harvested for FACS analysis by excitation at 405 nm (neutral pH) and 561 nm (acidic pH). **m** The relative mean mt-Keima 561/405 ratio was quantified by normalizing to DMSO group in cells treated as in **l**. Data shown represent the means ± SD of three biological replicates, **P < 0.01, ***P < 0.001, ****P < 0.0001; significance was determined by one-way ANOVA test followed by Tukey’s correction (**b**, **d**, **f**, **h**, **i**, **k**, **m**).

Having established that SMAD3 plays a positive regulatory role in PINK1 expression, we tried to assess the functional influence of SMAD3 on PINK1-dependent mitophagy. To do this, we studied the impact of the chemical inhibitor of SMAD3, E-SIS3, on mitochondrial protein degradation in cells treated with O/A. Reduction of mitochondrial proteins such as MFN1 (an OMM protein) and COXIV (an IMM protein) were assessed to evaluate the mitophagy level. We found that pre-treatment with E-SIS3 markedly reduced O/A-induced mitochondrial protein degradation (Fig. 4a, b; Supplementary Fig. S4a, b). Consistently, SMAD3 knockdown also abolished O/A-induced degradation of mitochondrial proteins (MFN1 and COXIV) (Fig. 4c, d; Supplementary Fig. S4c, d). Similar results were also found with the degradation of mitochondrial outer membrane protein TOMM20 examined by immunofluorescence (Fig. 4e, f). Since the recruitment of Parkin to damaged mitochondria is mediated by PINK1 as a critical step for mitophagy^10,24,36^, we utilized a previously established HeLa cells stably expressing mCherry-Parkin and mitoGFP to further examine whether SMAD3 could affect Parkin mitochondrial localization^37^. Indeed, the inhibition of SMAD3 effectively blocked Parkin translocation to mitochondria induced by O/A (Fig. 4g, h). Then, we examined the clearance of mtDNA, a well-known indicator of mitophagy^38–40^. By using qPCR to quantify the ratio of mtDNA to nuclear DNA, the clearance of mtDNA was markedly blocked in SMAD3-knockdown cells (Fig. 4i), indicating the defective mitophagy.

Moreover, we used mito-Keima assay, a well-established tool to monitor mitophagic flux and quantify mitophagy^41,42^. As shown in Fig. 4j, k and Supplementary Fig. S4e, upon O/A treatment, a remarkable gradual shift to longer-wavelength excitation was detected in the control cells, which was clearly reversed by either SMAD3 knockdown or SMAD3 inhibition with E-SIS3, indicating a decreased level of mitophagy upon SMAD3 suppression. Overall, the above data collectively suggest that SMAD3 promotes mitophagy.

### SMAD3 regulates *PINK1* transcription independent of TGFβ signaling

**Fig. 5:**
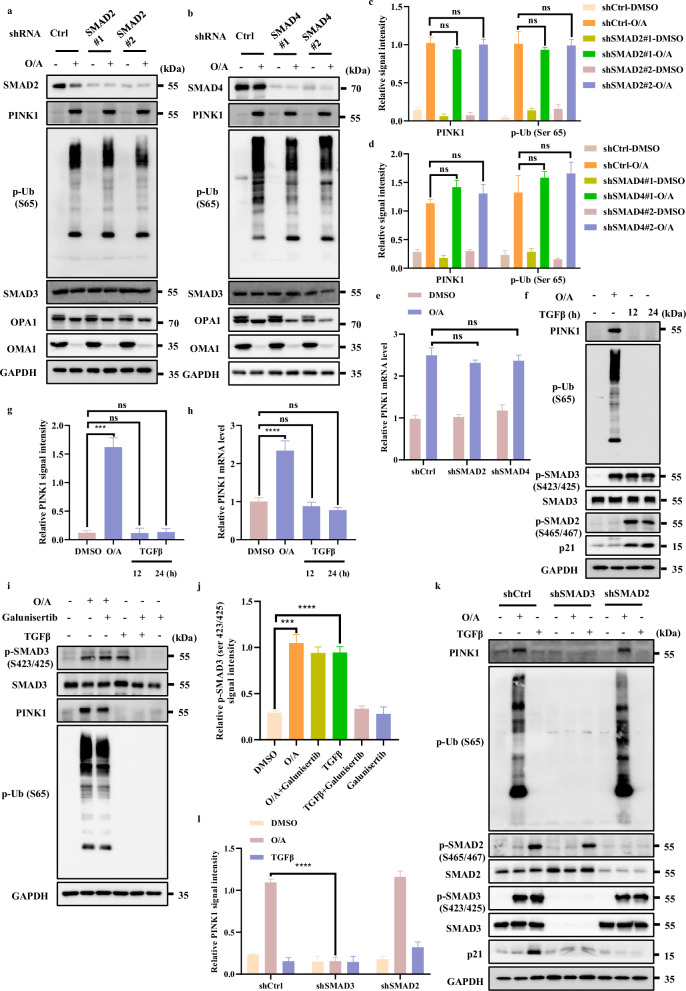
SMAD3 regulates *PINK1* transcription independent of TGFβ signaling. **a**, **b** SMAD2 (**a**) or SMAD4 (**b**) level was detected after infection with control or shSMAD2 or shSMAD4 lentiviral particles in HeLa WT cells. Cells were then incubated with OA (1/1 μM) for western blotting analysis with the indicated antibodies. **c** The indicated proteins were quantified by normalizing to GAPDH as loading control in cells treated as in **a**. **d** The indicated proteins were quantified by normalizing to GAPDH as loading control in cells treated as in **b**. **e** *PINK1* mRNA level was quantified using RT-PCR in HeLa WT shCtrl cells or HeLa WT shSMAD2 cells or HeLa WT shSMAD4 cells in the presence or absence of O/A (1/1 μM) for 4 h. **f** HeLa WT cells were treated with O/A (1/1 μM) for 4 h or TGFβ (100 ng/mL) for 12 h or 24 h and then collected for western blotting analysis with the indicated antibodies. **g** The indicated proteins were quantified by normalizing to GAPDH as loading control in cells treated as in **f**. **h** *PINK1* mRNA were analyzed in HeLa WT cells followed by O/A (1/1 μM) for 4 h or TGFβ (100 ng/mL) for 12 h or 24 h. **i** HeLa WT cells were pretreated with Galunisertib (20 μM) for 1 h. Subsequently, the cells were treated with or without O/A (1/1 μM) or TGFβ (100 ng/mL) for 4 h and subjected to western blotting analysis with the indicated antibodies. **j** The indicated proteins were quantified by normalizing to GAPDH as loading control in cells treated as in **i**. **k** HEK293T WT cells, HEK293T SMAD3-knockdown cells or HEK293T SMAD2-knockdown cells were treated with O/A (1/1 μM) or TGFβ (100 ng/mL) for 4 h and then subjected to western blotting analysis with the indicated antibodies. **l** The indicated proteins were quantified by normalizing to GAPDH as loading control in cells treated as in **k**. Data shown represent the means ± SD of three biological replicates, ***P < 0.001, ****P < 0.0001, ns no significance; significance was determined by one-way ANOVA test followed by Tukey’s correction (**c**–**e**, **g**, **h**, **j**, **l**).

It is well known that in the TGFβ signaling pathway, SMAD3 works together with other SMAD family members including SMAD2 and SMAD4 to control the transcription of downstream target genes^43^. To test whether other SMAD members are implicated in regulation of *PINK1* transcription, we knocked down SMAD2 and SMAD4 and found that both PINK1 and p-Ub (S65) levels were not affected upon mitochondrial damage (Fig. 5a‒d; Supplementary S5b‒d and f‒h), indicating that only SMAD3, but not SMAD2 or SMAD4, is involved in the regulation of *PINK1* transcription.

To further exclude the effect of SMAD2 or SMAD4 on MMP upon O/A treatment, we measured MMP upon SMAD2 or SMAD4 knockdown with/without O/A treatment. As shown in Supplementary Fig. S5a, e, the effect of O/A on MMP upon SMAD2 or SMAD4 knockdown is rather similar and comparable. Besides, the activation and degradation of OMA1 through its self-cleavage and its well-known substrate OPA1 caused by O/A-induced mitochondrial depolarization, were not affected by SMAD2 or SMAD4 knockdown (Fig. 5a, b). Thus, our data indicate that SMAD2 or SMAD4 knockdown does not alter mitochondrial depolarization induced by O/A treatment. In addition, we also found that *PINK1* mRNA level was not affected in cells with SMAD2 or SMAD4 knockdown (Fig. 5e), Therefore, it is believed that SMAD2 or SMAD4 has no regulatory role in the modulation of PINK1 expression.

Next, we examined whether TGFβ stimulation would affect *PINK1* transcription. As expected, the stimulation of TGFβ led to phosphorylation of SMAD2 and SMAD3, and increased the protein level of p21, a known downstream target of the TGFβ signaling pathway^44^, while TGFβ stimulation did not show any effect on PINK1 and p-Ub (Ser65) (Fig. 5f, g, Supplementary Fig. S5i, j). Consistently, TGFβ did not affect *PINK1* mRNA level (Fig. 5h, Supplementary Fig. S5j), suggesting that *PINK1* transcription is not regulated by the canonical TGFβ signaling pathway.

To further test the role of TGFβ signaling pathway in activation of SMAD3 caused by O/A, we utilized Galunisertib, a selective TGFβ-RI inhibitor^45^. As expected, we found that Galunisertib effectively abolished SMAD3 phosphorylation induced by TGFβ but failed to affect SMAD3 phosphorylation caused by O/A (Fig. 5i, j; Supplementary S5k). These findings thus suggest a non-canonical function of SMAD3 in control of *PINK1* transcription independent of TGFβ signaling.

Finally, we compared the different effects of TGFβ and O/A treatment on phosphorylation of SMAD2 and SMAD3. As shown in Fig. 5k, l, TGFβ caused an evident increase in SMAD2 and SMAD3 phosphorylation, accompanying by upregulation of p21 (Fig. 5k, l). In comparison, only SMAD3, but not SMAD2, was phosphorylated following O/A treatment (Fig. 5k, l). These data clearly suggest that O/A treatment specifically induces SMAD3 phosphorylation, but not affecting other components in the canonical TGFβ signaling pathway.

### SMAD3 is phosphorylated by PINK1

Since mitochondrial stress induces SMAD3 phosphorylation independent of TGFβ-R1 and PINK1 is a protein kinase, we speculated whether PINK1 could serve as a direct kinase for SMAD3. To test this hypothesis, we first utilized bioinformatics approaches to test the location of binding residue between human PINK1 (hPINK1) and SMAD3. The structure domain of PINK1 and SMAD3 are shown in Supplementary Fig. S6a. AlphaFold2 database was utilized to perform the structure prediction about Pediculus humanus corporis (human body louse) and Tribolium castaneum (Red flour beetle) PINK1. The Insertion-3 region (residues 188‒213) in the hPINK1 was known as a binding site for ubiquitin and was selected as the initial starting location for SMAD3 to bind^46^. For SMAD3, we focused on the protein binding structure MH2 domain regions that function in protein‒protein interaction while MH1 is responsible for direct DNA binding^47^. Therefore, MH2 domain of SMAD3 is strategically placed in proximity to hPINK1 (Supplementary Fig. S6a).

A total of 3.6 µs all atomistic molecular dynamics (MD) simulation was used for analyzing SMAD3‒PINK1 complex and MM/GBSA calculations. The average effective binding energy for nine different starting configurations was ‒45.2 ± 16.6 kcal mol^‒1^ for MH2 domains, with the highest effective binding energy structure achieved at ‒88.4 ± 10.0 kcal mol^‒1^ (Supplementary Fig. S6b). The residues E382 on SMAD3 and R326 on PINK1 contributed to 15.3% (13.5 ± 5.7 kcal mol^‒1^) of effective binding energy. Similarly, K377 on SMAD3 and D294 on PINK1 contributed 7.5% (6.6 ± 6.4 kcal mol^‒1^) to the effective binding energy.

**Fig. 6:**
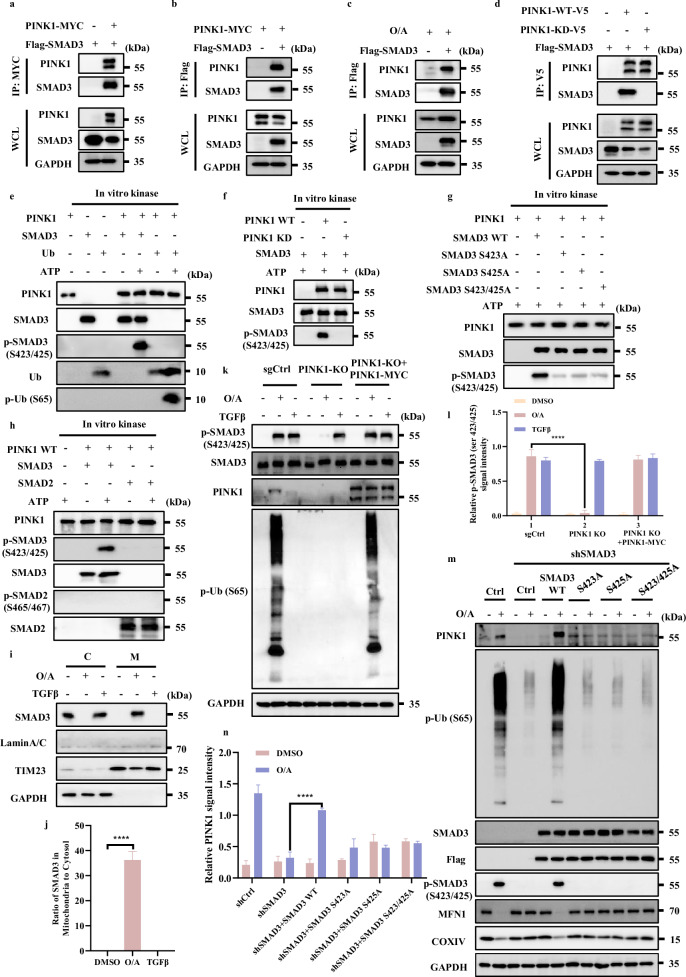
SMAD3 is phosphorylated by PINK1. **a** Lysates of HeLa WT cells overexpressing Flag-SMAD3 and/or PINK1-MYC were subjected to reciprocal co-IP to detect protein interaction. **b** Lysates of HeLa WT cells overexpressing PINK1-MYC and/or Flag-SMAD3 were subjected to reciprocal co-IP to detect protein interaction. **c** Lysates of HeLa WT cells were treated with/without O/A (1/1 μM) for 4 h followed by overexpressing Flag-SMAD3, and then subjected to reciprocal co-IP to detect protein interaction. **d** Lysates of HeLa WT cells overexpressing Flag-SMAD3 and PINK1-WT-V5/PINK1-KD-V5 were subjected to reciprocal co-IP to detect protein interaction. **e** SMAD3 and Ubiquitin (used as positive control substrate) phosphorylation by recombinant PINK1 were performed as described in Methods for further western blotting analysis with the indicated antibodies. **f** SMAD3 phosphorylation by recombinant wild-type PINK1 (WT PINK1) or kinase-dead mutant PINK1 (PINK1 KD) were performed as described in Methods for further western blotting analysis with the indicated antibodies. **g** The phosphorylation of SMAD3, SMAD3-S423A, SMAD3-S425A or SMAD3-S423/425A by recombinant PINK1 was performed as described in Methods for further western blotting analysis with the indicated antibodies. **h** SMAD2 and SMAD3 (used as positive control substrate) phosphorylation by recombinant PINK1 was performed as described in Methods for further western blotting analysis with the indicated antibodies. **i** Subcellular fractionation was performed to isolate the cytosolic and mitochondrial fractions in HeLa WT cells treated with O/A (1 μM) for 4 h. The samples were then analyzed by western blotting with the indicated antibodies. TIM23 and GAPDH were used as mitochondrial and cytosolic markers, respectively. **j** SMAD3 in mitochondria was quantified by normalizing to that in cytosol of cells treated as in **i**. **k** HeLa WT cells and HeLa WT PINK1-KO cells were treated with O/A (1/1 μM) or TGFβ (100 ng/mL) for 4 h in the presence or absence of PINK1-MYC transfection for 24 h and harvested at the indicated time points for western blotting analysis with the indicated antibodies. **l** The indicated proteins were quantified by normalizing to GAPDH as loading control in cells treated as in **k**. **m** HeLa YFP-Parkin WT and SMAD3 knockdown cells transfected with Flag-SMAD3, Flag-SMAD3 S423A, Flag-SMAD3 S425A or Flag-SMAD3 S423/425A for 24 h were treated with O/A (1/1 μM) or TGFβ (100 ng/mL) for 4 h and harvested at the indicated time points for western blotting analysis with the indicated antibodies. **n** The indicated proteins were quantified by normalizing to GAPDH as loading control in cells treated as in **m**. Data shown represent the means ± SD of three biological replicates, ****P < 0.0001; significance was determined by one-way ANOVA test followed by Tukey’s correction (**j**, **l**, **n**).

Next, we used co-immunoprecipitation assay to examine the protein‒protein interaction between PINK1 and SMAD3 and confirmed the interaction of PINK1 with SMAD3 in cells with or without Parkin expression, suggesting that this interaction is likely independent of Parkin (Fig. 6a, b; Supplementary Fig. S6c, d). Furthermore, endogenous PINK1 could also be immunoprecipitated by SMAD3 (Fig. 6c; Supplementary Fig. S6e). To further verify that the kinase activity of PINK1 was responsible for SMAD3 binding, we generated a PINK1 kinase-dead mutant (PINK1-KD, K219A/D362A/D384A) and found that the PINK1-KD failed to interact with SMAD3 (Fig. 6d), suggesting that the interaction between PINK1 and SMAD3 is dependent on the kinase activity of PINK1.

Second, we performed in vitro kinase assay to examine SMAD3 phosphorylation at its serine 423/425 that have been proposed as key sites for its activation^43^. Indeed, we found that PINK1 was able to directly phosphorylate SMAD3 at serine 423/425 (Fig. 6e). Increase of p-Ub was used a positive control of PINK1 kinase activity^11^. It is worth noticing that, PINK1-KD was unable to phosphorylate SMAD3 (Fig. 6f). This set of data thus clearly demonstrate that PINK1 is capable of phosphorylating SMAD3 at serine 423/425. Based on the fact that phosphorylation of SMAD3 at serine 423/425 is a key event in the TGFβ signaling pathway^43,48^, we further tested the specificity of PINK1-mediated SMAD3 phosphorylation by using their phosphorylation-defective mutants of SMAD3. Indeed, the mutant forms of S423/425 are much less responsive to PINK1 in vitro (Fig. 6g), confirming that PINK1 is capable of phosphorylating SMAD3 at S423/425.

Meanwhile, we tried to assess whether SMAD2 could also be phosphorylated by PINK1 at its serine 465/467, well-known sites for its phosphorylation and activation in the TGFβ signaling pathway^49^. Strikingly, only p-SMAD3 (serine 423/425), but not p-SMAD2 (serine 465/467) was detected (Fig. 6h), thus indicating that only SMAD3, but not SMAD2, is a direct substrate of PINK1 for phosphorylation.

After knowing the direct interaction and phosphorylation of SMAD3 by PINK1, we next attempted to explore whether SMAD3 is recruited to the damaged mitochondria. As shown in Fig. 6i, j, only in the presence of O/A-induced mitochondrial stress, but not TGFβ stimulation, SMAD3 exhibited a significant mitochondrial translocation. Such findings may help to explain the phenomenon that only O/A exposure, but not TGFβ treatment, caused a remarkable increase of *PINK1* mRNA and protein level even though both TGFβ and O/A could induce p-SMAD3.

Next, to further verify that SMAD3 phosphorylation observed in cells treated with O/A is dependent on PINK1, we generated *PINK1*-KO cells (Fig. 6k, l) and found that the phosphorylation of SMAD3 elicited by O/A treatment was markedly diminished in PINK1-KO cells, which could be restored by the reconstitution of PINK1 (Fig. 6k, l). Moreover, PINK1 did not show any effect on SMAD3 phosphorylation induced by TGFβ (Fig. 6k, l). Such results thus indicate that O/A-induced SMAD3 phosphorylation is PINK1-dependent while TGFβ-induced p-SMAD3 does not require PINK1.

Finally, to establish the role of SMAD3 phosphorylation in its regulation of *PINK1* transcription, we examined the effect of phosphorylation-defective mutants of SMAD3. Reconstitution of SMAD3-WT, but not its mutation forms in cells depleted of SMAD3, was able to restore *PINK1* mRNA (Supplementary Fig. S6g) and protein level following O/A treatment (Fig. 6m, n). Of note, p-SMAD3, stabilization of PINK1 and reduction of mitochondrial proteins (MFN1 and COXIV) were only found in cells with reconstitution of SMAD3-WT, but not in cells with overexpression of mutant SMAD3 (Fig. 6m, n). Similar data were found when mitophagy was detected using the mito-Keima assay (Supplementary Fig. S6h). Thus, data from this part of our study provide clear evidence that PINK1 is a kinase mediating SMAD3 phosphorylation at serine 423/425 both in vivo and in vitro, a process important for the transcriptional activity of SMAD3. Taken together, our results suggest the existence of a positive feedback loop between PINK1 and SMAD3 in response to mitochondrial damage.

### Activation of SMAD3 provides a pro-survival mechanism against mitochondrial stress

**Fig. 7:**
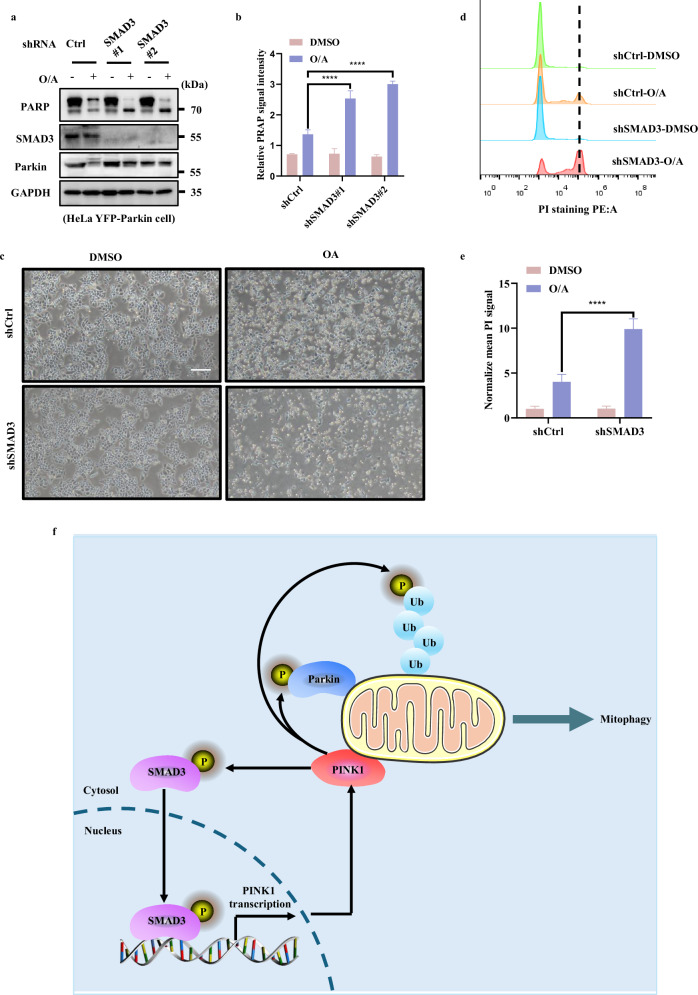
Activation of SMAD3 provides a pro-survival mechanism against mitochondrial stress. **a** HeLa YFP-Parkin WT and SMAD3-knockdown cells were treated with either DMSO or O/A (1 μM) for 24 h. The cells were then harvested for western blotting analysis with the indicated antibodies. **b** The indicated proteins were quantified by normalizing to GAPDH as loading control in cells treated as in **a**. **c** HeLa YFP-Parkin WT and SMAD3-knockdown cells were treated with either DMSO or O/A (1 μM) for 24 h. The cells were then imaged with phase contrast microscopy. Scale bar: 50 μm. **d** HeLa YFP-Parkin WT and SMAD3-knockdown cells treated as in **c** were stained with PI and subjected to FACS analysis. **e** The relative mean PE signaling was quantified by normalizing to DMSO group in cells treated as in **d**. **f** Schematic model for the positive regulatory role of SMAD3 in PINK1 and PINK1/Parkin-mediated mitophagy. Data shown represent the means ± SD of three biological replicates, ****P < 0.0001; significance was determined by one-way ANOVA test followed by Tukey’s correction (**b**, **e**).

It has been reported that PINK1 plays an important role in regulating cell survival upon mitochondrial depolarization^50–52^. Thus, we tried to evaluate how SMAD3 affected the susceptibility to cell death caused by mitochondrial depolarization. After knocking down SMAD3, apoptosis was monitored by analyzing CASP (caspase)-mediated PARP cleavage following O/A treatment. As shown in Fig. 7a, b and Supplementary Fig. S7a, b, f, the cleavage of PARP was greatly increased in SMAD3-knockdown cells. We then checked the cell viability of WT and SMAD3-knockdown cells under O/A treatment and found that SMAD3-depleted cells were much more sensitive to cell death compared to control cells (Fig. 7c; Supplementary Fig. S7c, g). The same results were further confirmed by the PI exclusion assay (Fig. 7d, e; Supplementary Fig. S7d, e, h), which showed that SMAD3-depleted cells were more sensitive to cell death induced by O/A. To clarify the type of cell death caused by SMAD3 inhibition under mitochondrial stress, we applied different cell death inhibitors such as caspase inhibitor Z-VAD-FMK, ferroptosis inhibitor-Ferrostatin-1 and necroptosis inhibitor-Necrostatin-1. As shown in Supplementary Fig. S7i, only Z-VAD-FMK can abolish the increased cleaved PARP stimulated by SMAD3 inhibition under mitochondrial stress. Thus, our data collectively suggest that SMAD3 mediates an anti-apoptotic response to mitochondrial damage, probably via activation of PINK1-mediated mitophagy.

## Discussion

In this study, we made two major findings. First, we found a novel non-canonical function of SMAD3 in transcriptional regulation of PINK1, a process independent of the TGFβ signaling pathway. Second, we found that PINK1 directly binds to and phosphorylates SMAD3 at serine 423/425, and this form of PTM promotes the transcriptional activity of SMAD3. Thus, we reveal a novel non-canonical regulatory role of SMAD3 in *PINK1* transcription and mitophagy. The activated PINK1 then targets SMAD3 for phosphorylation, which results in a positive feedforward loop in control of PINK1 expression and mitophagy (Fig. 7f).

PINK1 is an important serine-threonine kinase for the process of mitophagy, working upstream of the E3-ligase Parkin^53–55^. At present, most of the studies on regulation of PINK1 are focusing on its PTMs such as cleavage, ubiquitination and proteasomal degradation, while relatively much less is known about its transcriptional regulation. A recent report revealed the importance of intracellular localization of *PINK1* mRNA in control of its translation and expression and ultimately in regulation of mitophagy^56^, but without elaborating the transcription of PINK1 per se. Data from the current study demonstrating a novel function of SMAD3 in promoting mitophagy via regulation of *PINK1* transcription are indeed consistent with some of the earlier reports linking SMAD3 with mitochondrial function. For instance, it has been reported that SMAD3 deficiency impaired mitochondrial biogenesis through reducing oxidative enzyme activity^57^. In addition, SMAD3 could be degraded by LAMP2A-mediated CMA-lysosome pathway and impair mitochondrial biogenesis^58^. Moreover, SMAD3 has been identified as a key player to induce autophagy dysregulation through blocking TFEB-dependent lysosome biogenesis^59^. Another report showed that SMAD3 is involved in TGFβ-induced mitochondrial translocation of Parkin and mitophagy, without alteration of mitochondrial function^60^. Thus, all these findings suggest the possible participation of SMAD3 in regulation of mitophagy. Of note, FOXO3 was recognized as a transcriptional activator of PINK1 to mediate cell survival signaling^22^. Interestingly, FOXO3 and SMAD3 coordinately regulate the transcription of genes in a DNA-binding-dependent manner^61^. It would be of interest to examine whether SMAD3-mediated PINK1 transcriptional activation involves FOXO3 or other transcription factors (TFs). Thus, our data make a significant step forward by establishing a novel function of SMAD3 in promoting *PINK1* transcription.

SMAD3 is a well-known TGFβ effector required for transferring TGFβ signal into the nucleus with the coordination of SMAD2 and SMAD4^62–64^. In search of the molecular mechanisms underlying the regulation of *PINK1* transcription by SMAD3, we had a rather surprising finding that this function of SMAD3 is independent of SMAD2, SMAD4 and TGFβR1, indicating a non-canonical function of SMAD3 without involving the canonical TGFβ signaling pathway. Although it is genuinely believed that SMAD2 or SMAD4 is required for SMAD3’s function in TGFβ signaling pathway^65,66^, there are reports showing the functions of SMAD3 and SMAD2 independent of each other. For instance, chaperonin containing TCP1 subunit 6A (CCT6A) only binds to and inhibits SMAD2 but not SMAD3 to promote metastasis in lung cancer cells^67^, suggesting opposite functions of SMAD3 and SMAD2 in mediating lung cancer metastasis. Similarly, another study from the same group demonstrated that SMAD3 is capable of directly repressing the transcription of a series of cell cycle-promoting genes and consequently causes cellular senescence in lung epithelial cells, and this function of SMAD3 is independent of SMAD2 or SMAD4^68^. Therefore, it remains to be established whether SMAD3 has other partner to coordinate its nuclear colocalization in response to mitochondrial damage and transcriptional activation of PINK1.

Another important aspect of our study was the demonstration that O/A-induced mitochondria stress can activate SMAD3 by increasing SMAD3 phosphorylation at serine 423/425, same sites that are activated by the TGFβ signaling. In this study, via both in vitro and in vivo kinase assays we established that PINK1, a mitochondrial kinase, could directly phosphorylate SMAD3 serine 423/425. At present, only very limited reports available showing that SMAD3 could be phosphorylated by kinases other than TGFβ-R1 in the TGFβ signaling pathway. For example, p38 mitogen-activated protein kinase (MAPK) has been reported to mediate SMAD3 phosphorylation^69^. The decreased SMAD3 phosphorylation elicited by impaired MAPK is involved in PD pathology while PINK1 deficiency acted as a causative factor of autosomal recessive PD^34,70^.

In this study, we suggest that only SMAD3 phosphorylation by PINK1, but not by TGFβ, even at the same phosphorylation site, is capable of activating *PINK1* transcription. One possibility is that, as shown in Fig. 6i, j, only in the presence of O/A-induced mitochondrial stress, but not under TGFβ stimulation, SMAD3 exhibited a significant mitochondrial translocation. That is to say, even though both O/A and TGFβ could trigger SMAD3 phosphorylation at the same site, only O/A treatment can promote SMAD3 recruitment to the damaged mitochondria for further stabilization and activation by PINK1, which in turn, form a positive feedback loop through activating *PINK1* transcription. Moreover, our current data could not exclude other possibilities such as PINK1-mediated phosphorylation of SMAD3 at other sites which would lead to different preferences in control of its downstream target genes. Therefore, more work is needed to further establish the molecular mechanisms underlying PINK1-mediated SMAD3 phosphorylation.

Finally, we also attempted to establish the functional implication of this functional interaction between SMAD3 and PINK1 in control of cellular homeostasis in response to mitochondrial stress. Our data found that the feedback loop of SMAD3-PINK1 provides strong protective effects against cell death caused by mitochondrial stress although it needs more evidence to show the high susceptibility of SMAD3-KD cells is causatively linked to mitophagy. Taking the strong positive correlation between PINK1 and SMAD3 expression in human brain tissues (Fig. 1g‒j), we believe that SMAD3 could serve as a protective mechanism in the progression of PD. Future work should include quantification of SMAD3 expression level in specific brain regions or neuronal cells. Therefore, it might be of interest and importance to evaluate whether targeting the SMAD3-PINK1 loop could be used for the development of therapeutic approaches for mitochondria or mitophagy-related diseases.

Despite the novel discovery on the reciprocal effects between SMAD3 and PINK1, this study has several important limitations. First, it is not clear how SMAD3 works alone in regulation of *PINK1* transcription independent of the canonical TGFβ signaling pathway and independent of its usual partners such as SMAD2 and SMAD4. Second, the exact phosphorylation sites of SMAD3 by PINK1 remain to be further investigated. A comprehensive mass spectrometry (MS) analysis is needed to see whether there are any other phosphorylation sites in addition to S423/425. Third, this unique relationship between SMAD3 and PINK1 needs to be further evaluated using other cells such as primary neurons and in the context of in vivo disease models such as PD which is known to be closely associated with mitophagy defects.

Taking together, our findings highlight the presence of a positive feedback loop between SMAD3 and PINK1: SMAD3 promotes transcription of PINK1 and PINK1 in turn mediates SMAD3 phosphorylation and activation in response to mitochondrial stress. Understanding this novel regulatory mechanism provides a deeper insight into the pathological function of PINK1 and SMAD3 in the pathogenesis of neurodegenerative diseases such as PD.

## Materials and methods

### Cell culture

HeLa cells, HeLa YFP-Parkin cells, HEK293T cells and SY5Y cells were cultured in Dulbecco’s Modified Eagle Medium (DMEM, Gibco, 11965092) supplemented with 10% (v:v) fetal bovine serum (FBS, Thermo Fisher Scientific, Cat# 10270106), 100 U/mL penicillin (Gibco, Cat# 15140122) and 100 mg/mL streptomycin (Gibco, Cat# 15140122) at 37 °C in a humidified atmosphere containing 5% (v:v) CO_2_.

### Reagents and antibodies

#### Reagents used in this study

Antimycin A (Sigma-Aldrich, Cat# A8674), Oligomycin (Sigma-Aldrich, Cat# 11342), CCCP (Med Chem Express, Cat# HY-100941), propidium iodide (PI, Sigma-Aldrich, Cat# P4170), Actinomycin D (Sigma-Aldrich, Cat# A9415), Cycloheximide (Sigma, Cat# C4859), E-SIS3 (Med Chem Express, Cat# HY-13013), Galunisertib (Med Chem Express, Cat# HY-13226), Cisplatin (Med Chem Express, Cat# HY-17394), Z-VAD-FMK (Med Chem Express, Cat# HY-16658B), Necrostatin-1 (Med Chem Express, Cat# HY-15760), Ferrostatin-1 (Med Chem Express, Cat# HY-100579), Puromycin dihydrochloride (Med Chem Express, Cat# HY-B1743A), Anti-Flag Magnetic Beads (Med Chem Express, Cat# HY-K0207), Recombinant SMAD3 protein, Human (Flag-His) (Med Chem Express, Cat# HY-P71323), Recombinant P. humanus PINK1 Protein (R&D systems, Cat# AP-182-100), Recombinant SMAD3 protein (Med Chem Express, HY-P71323), Recombinant Human Ubiquitin Protein, CF (R&D systems, Cat# U-100H-10M).

#### Primary antibodies used in this study

anti-PINK1 (Cell Signaling Technology, Cat# 6946), anti-phospho-ubiquitin (Cell Signaling Technology, Cat# 62802s), anti-Parkin (Cell Signaling Technology, Cat# 4211s), anti-MFN1/mitofusin 1 (Cell Signaling Technology, Cat# 14739), anti-COXIV (Cell Signaling Technology, Cat# 4850), anti-MYC-tag (Cell Signaling Technology, Cat# 2276S), anti-OMA1 (Cell Signaling Technology, Cat# 95473S), anti-OPA1 (Cell Signaling Technology, Cat# 67589), anti-FLAG (Cell Signaling Technology, Cat# 14793), anti-SMAD3 (Cell Signaling Technology, Cat# 9523S), anti-p-SMAD3 (Cell Signaling Technology, Cat# 9520S), anti-SMAD2 (Cell Signaling Technology, Cat# 5339S), anti-p-SMAD2 (Cell Signaling Technology, Cat# 18338T), anti-SMAD4 (Cell Signaling Technology, Cat# 46535S), anti-p21 (Cell Signaling Technology, Cat# 2947s), anti-Lamin A/C (Cell Signaling Technology, Cat# 4777S), anti-PARP (Cell Signaling Technology, Cat# 9542S), anti-DNA (Progen Biotechnik, 61,014), anti-TOMM20 (Santa Cruz Technology, Cat# sc-17764), anti-TIM23 (Proteintech, Cat# 11123-1-AP), anti-GAPDH (Proteintech, Cat# 60004-1-Ig), anti-α-Tubulin (Proteintech, Cat# 11224-1-AP).

#### Secondary antibodies used in this study

Peroxidase-conjugated Affinity Pure Goat anti-mouse IgG (H + L), (Jackson Immuno Research, Cat# 115-035-146); Peroxidase-conjugated Affinity Pure Goat anti-Rabbit IgG (H + L), (Jackson Immuno Research, Cat# 111-035-144); Alexa Fluor-594 goat anti-mouse (Thermo fisher scientific, Cat# A-11032); Peroxidase-conjugated affinity pure goat anti-mouse IgG, light chain specific (Jackson Immuno Research, Cat# 115–035-174); Peroxidase-conjugated IgG fraction monoclonal mouse anti-rabbit, light chain specific (Jackson Immuno Research, Cat# 211-032-171).

### Plasmids and shRNA

Plasmids used in this study: PenCMV-3× Flag-SMAD3 (MiaoLingBio, China, Cat# P1198), pCMVTNT-PINK1 C-myc (Addgene, Cat# 13314); pLenti6-DEST PINK1-V5 WT (Addgene, Cat# 13320); pLenti6-DEST PINK1-V5 KD (Addgene, Cat# 13319), pLV-mitoGFP was a kind gift from Pantelis Tsoulfas (Addgene Cat# 44385); pBMN-mCherry-Parkin (Addgene Cat# 59419) was a kind gift from Richard Youle; pMD2.G-VSVg (Addgene Cat#12259); psPAX2 (Addgene, Cat# 12260); pLKO.1 (Addgene, Cat# 8453); lentiCRISPR v2 (Addgene, Cat# 52961).

PenCMV-3× Flag-SMAD3 S423A, PenCMV-3× Flag-SMAD3 S425A, PenCMV-3× Flag-SMAD3 S423/435A, PenCMV-3× Flag-PINK1 KD, were generated by the primers based on the vector of PenCMV-3× Flag-SMAD3 (MiaoLingBio, China, Cat# P1198).

3× Flag-SMAD3 S423A-F: 5’-GTTCCGCTGTGTCTTAGCTCGAGTCT-3’

3× Flag-SMAD3 S423A-R: 5’-ACACAGCGGAACAGCGGATGCTTGG-3’

3× Flag-SMAD3 S425A-F: 5’-GTGGCTTAGCTCGAGTCTAGAGGGC-3’

3× Flag-SMAD3 S425A-R: 5’-GAGCTAAGCCACACTGGAACAGCGGATGC-3’

3× Flag-SMAD3 S423/425A-F: 5’-GCTGTGGCTTAGCTCGAGTCTAGAGGG-3’

3× Flag-SMAD3 S423/425A-R: 5’-CTAAGCCACAGCGGAACAGCGGATGCTTGG -3’

3× Flag-PINK1 KD-F: 5’-CGCGGATCCAATTCGGTACCATGGCGG-3’

3× Flag-PINK1 KD-R: 5’-CTAGTCTAGAAGCTTCAGGGCTGCCCTCCATGA-3’

Site-directed mutagenesis constructs were confirmed by DNA sequencing.

The shRNA sequences against human *SMAD3*: shSMAD3#1, 5ʹ-TGAGCAGAACAGGTAGTATTA -3ʹ; and shSMAD3#2, 5ʹ-GAGCCTGGTCAAGAAACTCAA-3,

The shRNA sequences against human *SMAD4*: shSMAD4#1, 5ʹ-GTACTTCATACCATGCCGATT-3ʹ; and shSMAD4#2, 5ʹ-CGAGTTGTATCACCTGGAATT-3

The shRNA sequences against human *SMAD2*: shSMAD2#1, 5ʹ-TGGTGTTCAATCGCATACTAT -3ʹ; and shSMAD2#2, 5ʹ-CGATTAGATGAGCTTGAGAAA-3

They were synthesized and subcloned into the lentiviral vector pLKO.1 (Addgene, Cat# 8453) to drive the expression of shRNAs. Cells infected with lentivirus were selected with 2 µg/mL puromycin for 4 days in 10 cm dish.

### Western blotting

Western blotting was performed as previously described^71^. Briefly, cells were harvested with Lysis buffer (62.5 mM Tris-HCl, pH 6.8, 25% glycerol, 2% SDS, phosphatase inhibitor and proteinase inhibitor cocktail (Thermo Fisher Scientific, Cat# 78446), DTT (Thermo Fisher Scientific, Cat# R0861). Equal amounts of proteins by protein concentration determination Kit (Bio-Rad, Cat# 5000116) were loaded and separated on SDS-PAGE gels, and then transferred onto a PVDF membrane (Bio-Rad, Cat# 1620177). Membranes were then blocked by SuperBlock™ T20 (TBS) Blocking Buffer (Thermo Fisher Scientific, Cat# 37536) for 15 min, incubated overnight with primary antibodies, washed, and then incubated with secondary antibodies for 1 h at room temperature (RT). Detection was achieved using enhanced chemiluminescence (Millipore, Cat# WBLUR0500) and signals were visualized with the imager (Bio-Rad 170-8370 Touch Gel Documentation Imaging & Western Blot System).

### Co-IP assay

After the indicated transfection, all cells were suspended with Pierce™ IP Lysis Buffer (Thermo Fisher Scientific, Cat# 87787) together with phosphatase inhibitor and proteinase inhibitor cocktail (Thermo Fisher Scientific, Cat# 78446) and DTT (Thermo Fisher Scientific, Cat# R0861) for centrifugation at 14,000× *g* for 10 min at 4°. Part of the supernatant (2 mg) was transferred to a new tube as Input group. For FLAG IP or MYC IP, the remaining supernatant was incubated with either control IgG or anti-FLAG or anti-MYC overnight with gentle rotation at 4 °C. And then, Anti-c-Myc Magnetic Beads (Med Chem Express, Cat# HY-K0206) or Anti-Flag Magnetic Beads (Med Chem Express, Cat# HY-K0207) were subsequently added for additional gentle rotation for 4 h at RT. Next, the immunoprecipitants were washed with TBST buffer for 3 times and eluted by boiling for 10 min in 1× sample loading buffer (Bio-Rad Laboratories, Cat# 1610747) and analyzed on SDS-PAGE followed by western blotting analysis.

### Mito-Keima mitophagy assay

Mito-Keima mitophagy assay was performed as previously described^71^. Briefly, HeLa cells stably expressing YFP-Parkin harboring the mito-Keima vector were gifts from Dr. Jia-Hong Lu (University of Macau), which were infected with a lentivirus containing shSMAD3 and grown for several days. Next, cells were treated with or without OA in fresh growth medium for 8 h, and subsequently analyzed by flow cytometry.

### Generation of knockout cells

HeLa cells lacking PINK1 were generated using CRISPR/Cas9 gene editing. Briefly, specific DNA fragments targeting human *PINK1* DNA was subcloned into Lenti CRISPR plasmid (Addgene, Cat# 49535).

(Primer: F, 5’-CACCGTGGACCATCTGGTTCAACA-3´,

R, 5’-AAACTGTTGAACCAGATGGTCCAC-3´)

To produce lentivirus, recombinant Lenti-CRISPR was transfected in HEK293T cells together with plasmids psPAX2 and VSVG by PEI Max Transfection Grade Linear Polyethylenimine Hydrochloride (Equl, Cat# 24765-2). The medium containing lentiviral particles was collected and used for infecting HeLa cells. The infected cells were selected with puromycin (2 μg/mL) and grow into the single cells in 10 cm dish for the screen of *PINK1* knockout lines. The surviving clones were picked and selected by detecting *PINK1* mRNA level by Quantitative real-time PCR analysis.

PINK1 sense F: 5’-CAAGAGAGGTCCCAAGCAAC-3’

PINK1 anti-sense R: 5’-GGCAGCACATCAGGGTAGTC-3’

### Subcellular fractionation

Cells were harvested, pelleted by centrifugation at 1000× *g* for 3 min at 4 °C. We isolated the Nuclear and Cytoplasmic fraction by NE-PER™ Nuclear and Cytoplasmic Extraction Reagents kit (Thermo Fisher Scientific, Cat# 78835) or isolated the Mitochondrial and Cytoplasmic fraction by Mitochondria Isolation Kit for Cultured Cells (Thermo Fisher Scientific, Cat# 89874).

### Immunofluorescence staining

After the indicated treatment, the cells were fixed with 4% formaldehyde (Solarbio, Cat# P1110) for 15 min at RT. After being washed with PBS three times, the cells were blocked 10% BSA at RT for 0.5 h followed by the incubation with 0.1% Triton X-100 buffer for 15 min. Next, cells were incubated with the primary antibody overnight at 4° and washed with PBS buffer for three times. Subsequently, the cells were incubated with the secondary antibody for 1 h in the dark at RT. Finally, the cells were washed three times with PBS, and the slides were analyzed using a Leica confocal microscopy (Leica microsystem, Germany).

### RNA isolation and quantitative real-time PCR analysis

Total cellular RNA was extracted using PureLink RNA Mini Kit (Thermo Fisher Scientific, Cat# 12183025) following manufacturer’s instructions. cDNA synthesis was performed by iScript™ cDNA Synthesis Kit (Bio-Rad, Cat# 1708891) and then samples were subjected to real-time PCR by Bio-Rad CFX96 Touch Real-Time PCR Detection System. Relative expression of the mRNA was calculated by 2^−ΔΔCt^ method and normalized to *GAPDH*. The specific primers subjected to qPCR analysis are listed as follows:

PINK1-A sense F: 5’-CAAGAGAGGTCCCAAGCAAC-3’

PINK1-A anti-sense R: 5’-GGCAGCACATCAGGGTAGTC-3’

PINK1-B sense F: 5’-GCCTCATCGAGGAAAAACAGG-3’

PINK1-B anti-sense R: 5’-GTCTCGTGTCCAACGGGTC-3

PINK1-C sense F: 5’-CTGCCTTCCCCT TGGCC ATCAAG-3’

PINK1-C anti-sense R: 5’-GGGCTAGTTGCTTGGGACCTCTC-3

GAPDH sense F: 5’-GGAGCGAGATCCCTCCAAAAT-3’

GAPDH anti-sense R: 5’-GGCTGTTGTCATACTTCTCATGG-3’

Actin sense F: 5’-CATGTACGTTGCTATCCAGGC-3’

Actin anti-sense R: 5’-CTCCTTAATGTCACGCACGAT-3’

### Real-time PCR for quantitative detection of mtDNA

Detection of mtDNA was mentioned as described previously^72^. In brief, Total cellular genomic DNA was extracted using FastPure Cell/Tissue DNA Isolation Mini Kit (vazyme, Cat# DC102-01) following manufacturer’s instructions. Then, DNA samples were acted as templates and subjected to real-time PCR by Bio-Rad CFX96 Touch Real-Time PCR Detection System. Relative copy number of the mRNA was calculated by 2^−ΔΔCt^ method based on the forward primer (ND1-F) and reverse primer (ND1-R) for amplifying mtDNA gene *ND1* and normalization to human single-copy nuclear gene *β-globin*. The specific primers subjected to qPCR analysis are listed as follows:

ND1 sense F: 5’-CCCTAAAACCCGCCACATCT-3’

ND1 anti-sense F: 5’- GAGCGATGGTGAGAGCTAAGGT-3’

β-globin sense F: 5’- CTATGGGACGCTTGATGT-3’

β-globin antisense R: 5’- GCAATCATTCGTCTGTTT-3’

### Luciferase reporter assay

Human full-length PINK1 promoter-luciferase constructs were generated by the primers (5’- CTAGCTAGCGGAAGGGGGATTCAGATATGCC -3’, 5’-CCGCTCGAGTGCGCAGGCGCAGGCGCTGGT-3’) based on pGL3-Basic vector. The transcriptional regulation of *PINK1* promoters was measured after co-transfection of above cDNA or *PINK1-Δ1/2/3*, *PINK1-Δ1/2*, *PINK1-Δ1*, together with *pRL-TK* to normalize for transfection efficiencies. Luciferase activity was measured by Dual-Glo luciferase assay system (Promega, Cat# E2940) and tested by PerkinElmer Victor X3 Microplate Reader.

### ChIP assay

ChIP was performed by Simple ChIP® Plus Enzymatic Chromatin IP Kit (Cell Signalling Technology, Cat# 9005) according to the manufacture’s instruction. Briefly, collected cells were fixed with 1% formaldehyde for 10 min, quenched with 0.125 M glycine for 5 min at 37 °C and lysed in SDS Lysis Buffer. Cell lysate was sonicated to shear chromatin DNA to a size range of 200‒1000 bp. The supernatant was diluted in ChIP Dilution Buffer and precleared with 60 μL agarose beads for 30 min. The supernatant fraction was immunoprecipitated with primary antibody against SMAD3 overnight at 4 °C. Antibody‒chromatin complexes were pulled down with protein A agarose/salmon sperm DNA beads for 1 h at 4 °C. De-crosslinked DNA was subjected to qPCR analysis using specific primers as following:

PINK1-ARE-1-2-3-F

5’-CTAGCTAGCACGTATTCTCTCTGAAGCCTGCTAC-3’

PINK1-ARE-1-2-3-R

5’-CCGCTCGAGTGCGCAGGCGCAGGCGCTGGT-3’

PINK1-ARE2-3-F

5’- CTAGCTAGCATATATCCAATTTCACCTTTTTGGGATACC-3’

PINK1-ARE-2-3-R

5’-CCGCTCGAGTGCGCAGGCGCAGGCGCTGGT-3’

PINK1-ARE-3-F

5’-CTAGCTAGCCAGGCTGTGAGCTACCACACCC-3’

PINK1-ARE-3-R

5’-CCGCTCGAGTGCGCAGGCGCAGGCGCTGGT-3’

### In vitro protein kinase assay

2 μg Recombinant P. humanus PINK1 Protein (R&D systems, Cat# AP-182-100) and 1 μg Recombinant SMAD3 protein (Med Chem Express, Cat# HY-P71323) or 1 μg Recombinant Human Ubiquitin Protein, CF (R&D systems, Cat# U-100H-10M) were mixed with kinase buffer (30 mM HEPES, 50 mM potassium acetate, 5 mM MgCl_2_), and then incubated at 30 °C for 2 h in the presence of cold ATP (500 μM). The reaction mixture was terminated with 2× SDS loading buffer and subjected to SDS-PAGE.

3× Flag-tagged SMAD3 S423A, 3× Flag-tagged SMAD3 S425A, 3× Flag-tagged SMAD3 S423/425A protein were purified from HEK293T SMAD3 knockout cells transiently transfected with 3× Flag-tagged SMAD3 S423A, 3× Flag-tagged SMAD3 S425A, 3× Flag-tagged SMAD3 S423/425A plasmid for 24 h.

3× Flag-tagged PINK1 KD protein was purified from HEK293T *PINK1*-KO cells transiently transfected with 3× Flag-tagged *PINK1*-KD plasmid for 24 h.

Then cells were lysed with RIPA buffer and subjected to immunoprecipitation with Anti-Flag Magnetic Beads (Med Chem Express, Cat# HY-K0207). Samples were washed 4 times with TBST and eluted with Flag peptide (50 µg/mL, Sigma-Aldrich, Cat# F4799) for 2 h at RT. The eluted proteins were subjected to kinase reaction in the kinase buffer in the presence of ATP for 2 h at 30° and subjected to SDS-PAGE and immunoblotting.

### The MD simulations for protein-protein binding

The predicted human PINK1 (hPINK1) protein crystal structure model was obtained from the AlphaFold2 database^73^. The Alphafold2 model showed the N-terminus model confidence is low (pLDDT < 50) and we used the Chimera package to remove the tail regions, with the remainder model containing residues from 101–581 aa^74^. For the SMAD3 protein, we used the MH2 (PDB ID: 1MJS, resolution 1.91 Å, residues 228–424) domain from the protein database (https://www.rcsb.org/, accessed 23rd November 2023). The MODELLER software in the Chimera package was used to fix the missing atoms in the SMAD3 PDB files by generating 10 predicted models^75^. We position the SMAD3 in close proximity to the hPINK1 protein insertion-3 region (residues 188–213)^46^.

Following the docking protocols of the RosettaDock 5.0 package, we generated the approximation position of SMAD3 and hPINK1^76^. Briefly, the relax and backrub protocols in the package were utilized for the initial low-resolution prediction to generate 100 structures for each protein. The information was relayed to the high-resolution docking protocols, where the beta_nova16 energy function was applied. For each system, 50,000 iterations were used to acquire a global search for the interaction site between the proteins. We used the highest 9 interface score (I_sc) as the basis for determining the initial 9 protein structure configurations for protein–protein binding.

The MD simulations were computed by the GROMACS v.2023 software^77^. The selected 9 configurations generated were used as the starting position of MD simulations and the AMBER14sb forcefield was employed for modeling the protein complex and ions^78^. For each system, the protein complex is situated at the center of the simulation box and the periodic boundaries were set at 1.5 nm away from the protein complex. Then the system is saturated with TIP3P water and neutralized by Cl^–^ ions, with the concentration of salt ions set at 0.15 mol/L. The steepest descent algorithm was first performed and followed by a 1.2 ns equilibration run. During the equilibration run, six temperature annealing points ranging from 270 K to 310 K were used to relax the system. Berendsen pressure coupling and temperature coupling were used to regulate 1 bar and the annealing temperature in the equilibration run^79^. Subsequently, a 200 ns production run was simulated per system, with Parrinello-Rahman pressure coupling and V-rescale temperature coupling to maintain the conditions as 1 bar and 310 K^80^. The Particle Mesh Ewald (PME) method treated the long-range electrostatic potentials, and the cut-off distance was set at 0.8 nm. Leap-frog algorithms were used for integrating Newton’s equation of motion with a 2-fs time step. LINC algorithms were used to constrain hydrogen bonds at equilibrium length^81^. The trajectories for each system were used as the input of MM/GBSA and the calculations were performed by gmx MMPBSA software^82^. Only the final 100 ns (5000 frames) were used for calculating the effective binding energy between the protein. The implicit solvent model was approximated by the modified generalized Born model and the ion radius was approximated by using the “mbondi2”^83,84^.

### Cell death measured by PI staining

PI (Sigma-Aldrich, Cat# P4170) is a red-fluorescent DNA dye which penetrates damaged cellular membranes and used for the determination of cell viability. Briefly, after cell plated in six-well plates and treatment finished, cells were collected and suspended in cold PBS buffer. Then, cells were stained with PI and detected by flow cytometry.

### Statistical analysis

The statistical significance of the mean differences observed between two samples was determined by the student's two-tailed *t*-test. The means of more than 3 samples were performed by One-way ANOVA with Dunnett’s multiple comparisons test, whereas Two-way ANOVA with Sidak’s multiple comparisons test was utilized to compare multiple groups of two factors using GraphPad Prism 8. Data are shown as means ± SD. of the results from 3 independent experiments. Statistical significance is shown as *P < 0.05, **P < 0.01, ***P < 0.001, ****P < 0.0001.

## Supplementary information

Supplementary figures

## References

[1] Georgakopoulos, N. D., Wells, G. & Campanella, M. The pharmacological regulation of cellular mitophagy. *Nat. Chem. Biol.* **13**, 136–146 (2017). 28103219 10.1038/nchembio.2287

[2] Youle, R. J. & Narendra, D. P. Mechanisms of mitophagy. *Nat. Rev. Mol. Cell Biol.* **12**, 9–14 (2011). 21179058 10.1038/nrm3028 PMC4780047

[3] Lu, G. et al. Autophagy in health and disease: From molecular mechanisms to therapeutic target. *MedComm.* **3**, e150 (2022). 35845350 10.1002/mco2.150 PMC9271889

[4] Pickles, S., Vigie, P. & Youle, R. J. Mitophagy and quality control mechanisms in mitochondrial maintenance. *Curr. Biol.* **28**, R170–R185 (2018). 29462587 10.1016/j.cub.2018.01.004 PMC7255410

[5] Wang, L., Qi, H., Tang, Y. & Shen, H. M. Post-translational modifications of key machinery in the control of mitophagy. *Trends Biochem. Sci.* **45**, 58–75 (2020). 31606339 10.1016/j.tibs.2019.08.002

[6] Deas, E. et al. PINK1 cleavage at position A103 by the mitochondrial protease PARL. *Hum. Mol. Genet.* **20**, 867–879 (2011). 21138942 10.1093/hmg/ddq526 PMC3033179

[7] Greene, A. W. et al. Mitochondrial processing peptidase regulates PINK1 processing, import and Parkin recruitment. *EMBO Rep.* **13**, 378–385 (2012). 22354088 10.1038/embor.2012.14 PMC3321149

[8] Jin, S. M. et al. Mitochondrial membrane potential regulates PINK1 import and proteolytic destabilization by PARL. *J. Cell Biol.* **191**, 933–942 (2010). 21115803 10.1083/jcb.201008084 PMC2995166

[9] Yamano, K. & Youle, R. J. PINK1 is degraded through the N-end rule pathway. *Autophagy* **9**, 1758–1769 (2013). 24121706 10.4161/auto.24633 PMC4028335

[10] Narendra, D. P. et al. PINK1 is selectively stabilized on impaired mitochondria to activate Parkin. *PLoS Biol.* **8**, e1000298 (2010). 20126261 10.1371/journal.pbio.1000298 PMC2811155

[11] Kane, L. A. et al. PINK1 phosphorylates ubiquitin to activate Parkin E3 ubiquitin ligase activity. *J. Cell Biol.* **205**, 143–153 (2014). 24751536 10.1083/jcb.201402104 PMC4003245

[12] Koyano, F. et al. Ubiquitin is phosphorylated by PINK1 to activate parkin. *Nature* **510**, 162–166 (2014). 24784582 10.1038/nature13392

[13] Kazlauskaite, A. et al. Parkin is activated by PINK1-dependent phosphorylation of ubiquitin at Ser65. *Biochem. J* **460**, 127–139 (2014). 24660806 10.1042/BJ20140334 PMC4000136

[14] Shiba-Fukushima, K. et al. PINK1-mediated phosphorylation of the Parkin ubiquitin-like domain primes mitochondrial translocation of Parkin and regulates mitophagy. *Sci. Rep.* **2**, 1002 (2012). 23256036 10.1038/srep01002 PMC3525937

[15] Iguchi, M. et al. Parkin-catalyzed ubiquitin-ester transfer is triggered by PINK1-dependent phosphorylation. *J. Biol. Chem.* **288**, 22019–22032 (2013). 23754282 10.1074/jbc.M113.467530 PMC3724655

[16] Kondapalli, C. et al. PINK1 is activated by mitochondrial membrane potential depolarization and stimulates Parkin E3 ligase activity by phosphorylating Serine 65. *Open Biol.* **2**, 120080 (2012). 22724072 10.1098/rsob.120080 PMC3376738

[17] Okatsu, K. et al. Phosphorylated ubiquitin chain is the genuine Parkin receptor. *J. Cell Biol.* **209**, 111–128 (2015). 25847540 10.1083/jcb.201410050 PMC4395490

[18] Ordureau, A. et al. Defining roles of PARKIN and ubiquitin phosphorylation by PINK1 in mitochondrial quality control using a ubiquitin replacement strategy. *Proc. Natl. Acad. Sci. USA* **112**, 6637–6642 (2015). 25969509 10.1073/pnas.1506593112 PMC4450373

[19] Ordureau, A. et al. Quantitative proteomics reveal a feedforward mechanism for mitochondrial PARKIN translocation and ubiquitin chain synthesis. *Mol. Cell* **56**, 360–375 (2014). 25284222 10.1016/j.molcel.2014.09.007 PMC4254048

[20] Shiba-Fukushima, K. et al. Phosphorylation of mitochondrial polyubiquitin by PINK1 promotes Parkin mitochondrial tethering. *PLoS Genet* **10**, e1004861 (2014). 25474007 10.1371/journal.pgen.1004861 PMC4256268

[21] Wang, M. et al. The emerging multifaceted role of PINK1 in cancer biology. *Cancer Sci.* **113**, 4037–4047 (2022). 36071695 10.1111/cas.15568 PMC9746061

[22] Mei, Y. et al. FOXO3a-dependent regulation of Pink1 (Park6) mediates survival signaling in response to cytokine deprivation. *Proc. Natl. Acad. Sci. USA* **106**, 5153–5158 (2009). 19276113 10.1073/pnas.0901104106 PMC2654023

[23] Soutar, M. P. M. et al. Regulation of mitophagy by the NSL complex underlies genetic risk for Parkinson’s disease at 16q11.2 and MAPT H1 loci. *Brain* **145**, 4349–4367 (2022). 36074904 10.1093/brain/awac325 PMC9762952

[24] Vives-Bauza, C. et al. PINK1-dependent recruitment of Parkin to mitochondria in mitophagy. *Proc. Natl Acad. Sci. USA* **107**, 378–383 (2010). 19966284 10.1073/pnas.0911187107 PMC2806779

[25] Crowley, L. C., Christensen, M. E. & Waterhouse, N. J. Measuring mitochondrial transmembrane potential by TMRE staining. *Cold Spring Harb. Protoc.* **2016**, 1092–1096 (2016). 10.1101/pdb.prot087361 27934682

[26] Baker, M. J. et al. Stress-induced OMA1 activation and autocatalytic turnover regulate OPA1-dependent mitochondrial dynamics. *EMBO J.* **33**, 578–593 (2014). 24550258 10.1002/embj.201386474 PMC3989652

[27] Xie, X., Rigor, P. & Baldi, P. MotifMap: a human genome-wide map of candidate regulatory motif sites. *Bioinformatics* **25**, 167–174 (2009). 19017655 10.1093/bioinformatics/btn605 PMC2732295

[28] Hasson, S. A. et al. High-content genome-wide RNAi screens identify regulators of parkin upstream of mitophagy. *Nature* **504**, 291–295 (2013). 24270810 10.1038/nature12748 PMC5841086

[29] Hill, C. S. Transcriptional Control by the SMADs. *Cold Spring Harb. Perspect. Biol.* **8**, a022079 (2016). 27449814 10.1101/cshperspect.a022079 PMC5046698

[30] Kurisaki, A. et al. The mechanism of nuclear export of Smad3 involves exportin 4 and Ran. *Mol. Cell. Biol.* **26**, 1318–1332 (2006). 16449645 10.1128/MCB.26.4.1318-1332.2006 PMC1367208

[31] Shi, Y. & Massague, J. Mechanisms of TGF-beta signaling from cell membrane to the nucleus. *Cell* **113**, 685–700 (2003). 12809600 10.1016/s0092-8674(03)00432-x

[32] Javelaud, D. & Mauviel, A. Mammalian transforming growth factor-betas: Smad signaling and physio-pathological roles. *Int. J. Biochem. Cell Biol.* **36**, 1161–1165 (2004). 15109563 10.1016/S1357-2725(03)00255-3

[33] Jinnin, M., Ihn, H. & Tamaki, K. Characterization of SIS3, a novel specific inhibitor of Smad3, and its effect on transforming growth factor-beta1-induced extracellular matrix expression. *Mol. Pharm.* **69**, 597–607 (2006). 10.1124/mol.105.017483 16288083

[34] Liu, Y., Yu, L., Xu, Y., Tang, X. & Wang, X. Substantia nigra Smad3 signaling deficiency: relevance to aging and Parkinson’s disease and roles of microglia, proinflammatory factors, and MAPK. *J. Neuroinflammation* **17**, 342 (2020). 33198771 10.1186/s12974-020-02023-9 PMC7670688

[35] Huang, Z. et al. (E)-SIS3 suppressed osteosarcoma progression via promoting cell apoptosis, arresting cell cycle, and regulating the tumor immune microenvironment. *Drug Dev. Res.* **84**, 1751–1763 (2023). 10.1002/ddr.22120 37784254

[36] Narendra, D., Tanaka, A., Suen, D. F. & Youle, R. J. Parkin is recruited selectively to impaired mitochondria and promotes their autophagy. *J. Cell Biol.* **183**, 795–803 (2008). 19029340 10.1083/jcb.200809125 PMC2592826

[37] Tan, H. W. S. et al. A degradative to secretory autophagy switch mediates mitochondria clearance in the absence of the mATG8-conjugation machinery. *Nat. Commun.* **13**, 3720 (2022). 35764633 10.1038/s41467-022-31213-7 PMC9240011

[38] Wang, L. et al. PTEN-L is a novel protein phosphatase for ubiquitin dephosphorylation to inhibit PINK1-Parkin-mediated mitophagy. *Cell Res.* **28**, 787–802 (2018). 29934616 10.1038/s41422-018-0056-0 PMC6082900

[39] Yi, J. et al. Spautin-1 promotes PINK1-PRKN-dependent mitophagy and improves associative learning capability in an alzheimer disease animal model. *Autophagy*, **20**, 2655–2676 (2024). 10.1080/15548627.2024.2383145 PMC11587853 39051473

[40] Lazarou, M. et al. The ubiquitin kinase PINK1 recruits autophagy receptors to induce mitophagy. *Nature* **524**, 309–314 (2015). 26266977 10.1038/nature14893 PMC5018156

[41] Sun, N. et al. A fluorescence-based imaging method to measure in vitro and in vivo mitophagy using mt-Keima. *Nat. Protoc.* **12**, 1576–1587 (2017). 28703790 10.1038/nprot.2017.060

[42] Sun, N. et al. Measuring in vivo mitophagy. *Mol. Cell* **60**, 685–696 (2015). 26549682 10.1016/j.molcel.2015.10.009 PMC4656081

[43] Heldin, C. H., Miyazono, K. & ten Dijke, P. TGF-beta signalling from cell membrane to nucleus through SMAD proteins. *Nature* **390**, 465–471 (1997). 9393997 10.1038/37284

[44] Lasfar, A. & Cohen-Solal, K. A. Resistance to transforming growth factor beta-mediated tumor suppression in melanoma: are multiple mechanisms in place? *Carcinogenesis* **31**, 1710–1717 (2010). 20656791 10.1093/carcin/bgq155 PMC2981460

[45] Herbertz, S. et al. Clinical development of galunisertib (LY2157299 monohydrate), a small molecule inhibitor of transforming growth factor-beta signaling pathway. *Drug Des. Devel. Ther.* **9**, 4479–4499 (2015). 10.2147/DDDT.S86621 PMC4539082 26309397

[46] Gan, Z. Y. et al. Activation mechanism of PINK1. *Nature* **602**, 328–335 (2022). 34933320 10.1038/s41586-021-04340-2 PMC8828467

[47] Tzavlaki, K. & Moustakas, A. TGF-beta Signaling. *Biomolecules* **10**, 487 (2020). 10.3390/biom10030487 PMC7175140 32210029

[48] Ooshima, A., Park, J. & Kim, S. J. Phosphorylation status at Smad3 linker region modulates transforming growth factor-beta-induced epithelial-mesenchymal transition and cancer progression. *Cancer Sci.* **110**, 481–488 (2019). 30589983 10.1111/cas.13922 PMC6361575

[49] Abdollah, S. et al. TbetaRI phosphorylation of Smad2 on Ser465 and Ser467 is required for Smad2-Smad4 complex formation and signaling. *J. Biol. Chem.* **272**, 27678–27685 (1997). 9346908 10.1074/jbc.272.44.27678

[50] Arena, G. et al. PINK1 protects against cell death induced by mitochondrial depolarization, by phosphorylating Bcl-xL and impairing its pro-apoptotic cleavage. *Cell Death Differ.* **20**, 920–930 (2013). 23519076 10.1038/cdd.2013.19 PMC3679455

[51] Yoshii, S. R., Kishi, C., Ishihara, N. & Mizushima, N. Parkin mediates proteasome-dependent protein degradation and rupture of the outer mitochondrial membrane. *J. Biol. Chem.* **286**, 19630–19640 (2011). 21454557 10.1074/jbc.M110.209338 PMC3103342

[52] Liang, J. R. et al. USP30 deubiquitylates mitochondrial Parkin substrates and restricts apoptotic cell death. *EMBO Rep.* **16**, 618–627 (2015). 25739811 10.15252/embr.201439820 PMC4428036

[53] Shimura, H. Familial Parkinson disease gene product, parkin, is a ubiquitin-protein ligase. *Nat. Genet.* **25**, 302–305 (2000). 10888878 10.1038/77060

[54] da Costa, C. A. et al. Transcriptional repression of p53 by parkin and impairment by mutations associated with autosomal recessive juvenile Parkinson’s disease. *Nat. Cell Biol.* **11**, 1370–1375 (2009). 19801972 10.1038/ncb1981 PMC2952934

[55] Alves da Costa, C., Duplan, E., Rouland, L. & Checler, F. The transcription factor function of Parkin: breaking the dogma. *Front. Neurosci.* **12**, 965 (2018). 30697141 10.3389/fnins.2018.00965 PMC6341214

[56] Hees, J. T. et al. Insulin signalling regulates Pink1 mRNA localization via modulation of AMPK activity to support PINK1 function in neurons. *Nat. Metab.* **6**, 514–530 (2024). 38504131 10.1038/s42255-024-01007-w PMC10963278

[57] Ge, X. et al. Lack of Smad3 signaling leads to impaired skeletal muscle regeneration. *Am. J. Physiol. Endocrinol. Metab.* **303**, E90–102, (2012). 22535746 10.1152/ajpendo.00113.2012

[58] Liu, H. et al. Chaperone-mediated autophagy regulates cell growth by targeting SMAD3 in glioma. *Neurosci. Bull.* **38**, 637–651 (2022). 35267139 10.1007/s12264-022-00818-9 PMC9206062

[59] Yang, C. et al. SMAD3 promotes autophagy dysregulation by triggering lysosome depletion in tubular epithelial cells in diabetic nephropathy. *Autophagy* **17**, 2325–2344 (2021). 33043774 10.1080/15548627.2020.1824694 PMC8496726

[60] Lee, J. H. et al. Parkin-mediated mitophagy by TGF-beta is connected with hepatic stellate cell activation. *Int. J. Mol. Sci.* **24**, 14826 (2023). 37834275 10.3390/ijms241914826 PMC10573240

[61] Bollinger, L. M., Witczak, C. A., Houmard, J. A. & Brault, J. J. SMAD3 augments FoxO3-induced MuRF-1 promoter activity in a DNA-binding-dependent manner. *Am. J. Physiol. Cell Physiol.* **307**, C278–287, (2014). 24920680 10.1152/ajpcell.00391.2013 PMC4121583

[62] Neuzillet, C. et al. Targeting the TGFbeta pathway for cancer therapy. *Pharm. Ther.* **147**, 22–31 (2015). 10.1016/j.pharmthera.2014.11.001 25444759

[63] Meng, X. M., Nikolic-Paterson, D. J. & Lan, H. Y. TGF-beta: the master regulator of fibrosis. *Nat. Rev. Nephrol.* **12**, 325–338 (2016). 27108839 10.1038/nrneph.2016.48

[64] Ikushima, H. & Miyazono, K. TGFbeta signalling: a complex web in cancer progression. *Nat. Rev. Cancer* **10**, 415–424 (2010). 20495575 10.1038/nrc2853

[65] Hata, A. & Chen, Y. G. TGF-beta signaling from receptors to Smads. *Cold Spring Harb. Perspect. Biol*. **8**, 10.1101/cshperspect.a022061 (2016). 10.1101/cshperspect.a022061 PMC5008074 27449815

[66] Budi, E. H., Duan, D. & Derynck, R. Transforming growth factor-beta receptors and Smads: regulatory complexity and functional versatility. *Trends Cell Biol.* **27**, 658–672 (2017). 28552280 10.1016/j.tcb.2017.04.005

[67] Ying, Z. et al. CCT6A suppresses SMAD2 and promotes prometastatic TGF-beta signaling. *J. Clin. Invest.* **127**, 1725–1740 (2017). 28375158 10.1172/JCI90439 PMC5409794

[68] Liu, X. et al. ATOH8 binds SMAD3 to induce cellular senescence and prevent Ras-driven malignant transformation. *Proc. Natl. Acad. Sci. USA* **120**, e2208927120 (2023). 10.1073/pnas.2208927120 PMC9934021 36626550

[69] Furukawa, F. et al. p38 MAPK mediates fibrogenic signal through Smad3 phosphorylation in rat myofibroblasts. *Hepatology* **38**, 879–889 (2003). 14512875 10.1053/jhep.2003.50384

[70] Pickrell, A. M. & Youle, R. J. The roles of PINK1, parkin, and mitochondrial fidelity in Parkinson’s disease. *Neuron* **85**, 257–273 (2015). 25611507 10.1016/j.neuron.2014.12.007 PMC4764997

[71] Lu, G. et al. WIPI2 positively regulates mitophagy by promoting mitochondrial recruitment of VCP. *Autophagy* **18**, 2865–2879 (2022). 35389758 10.1080/15548627.2022.2052461 PMC9673930

[72] Gahan, M. E. et al. Quantification of mitochondrial DNA in peripheral blood mononuclear cells and subcutaneous fat using real-time polymerase chain reaction. *J. Clin. Virol.* **22**, 241–247 (2001). 11564588 10.1016/s1386-6532(01)00195-0

[73] Jumper, J. et al. Highly accurate protein structure prediction with AlphaFold. *Nature* **596**, 583–589 (2021). 34265844 10.1038/s41586-021-03819-2 PMC8371605

[74] Meng, E. C., Pettersen, E. F., Couch, G. S., Huang, C. C. & Ferrin, T. E. Tools for integrated sequence-structure analysis with UCSF Chimera. *BMC Bioinforma.* **7**, 339 (2006). 10.1186/1471-2105-7-339 PMC1570152 16836757

[75] Šali, A. & Blundell, T. L. Comparative protein modelling by satisfaction of spatial restraints. *J. Mol. Biol.* **234**, 779–815 (1993). 8254673 10.1006/jmbi.1993.1626

[76] Lyskov, S. & Gray, J. J. The RosettaDock server for local protein–protein docking. *Nucleic Acids Res.* **36**, W233–W238 (2008). 18442991 10.1093/nar/gkn216 PMC2447798

[77] Berendsen, H. J. C., van der Spoel, D. & van Drunen, R. GROMACS: A message-passing parallel molecular dynamics implementation. *Comput. Phys. Commun.* **91**, 43–56 (1995).

[78] Maier, J. A. et al. ff14SB: improving the accuracy of protein side chain and backbone parameters from ff99SB. *J. Chem. Theory Comput.* **11**, 3696–3713 (2015). 26574453 10.1021/acs.jctc.5b00255 PMC4821407

[79] Berendsen, H. J. C., Postma, J. P. M., van Gunsteren, W. F., DiNola, A. & Haak, J. R. Molecular dynamics with coupling to an external bath. *J. Chem. Phys.* **81**, 3684–3690 (1984).

[80] Parrinello, M. & Rahman, A. Polymorphic transitions in single crystals: A new molecular dynamics method. *Int. J. Appl. Phys.* **52**, 7182–7190 (1981).

[81] Hess, B., Bekker, H., Berendsen, H. J. C. & Fraaije, J. G. E. M. LINCS: A linear constraint solver for molecular simulations. *J. Comput. Chem.* **18**, 1463–1472 (1997).

[82] Valdés-Tresanco, M. S., Valdés-Tresanco, M. E., Valiente, P. A. & Moreno, E. gmx_MMPBSA: a new tool to perform end-state free energy calculations with GROMACS. *J. Chem. Theory Comput.* **17**, 6281–6291 (2021). 34586825 10.1021/acs.jctc.1c00645

[83] Onufriev, A., Bashford, D. & Case, D. A. Exploring protein native states and large-scale conformational changes with a modified generalized born model. *Proteins: Struct. Funct. Bioinforma.* **55**, 383–394 (2004). 10.1002/prot.20033 15048829

[84] Nunes, R., Vila-Viçosa, D. & Costa, P. J. Tackling halogenated species with PBSA: effect of emulating the σ-hole. *J. Chem. Theory Comput.* **15**, 4241–4251 (2019). 31142112 10.1021/acs.jctc.9b00106

