# Supplementary material for: A positive feedback loop between SMAD3 and PINK1 in regulation of mitophagy
Source: Cell Discov. 2025 Mar 11;11:22. doi: 10.1038/s41421-025-00774-4 (PMC11894195; doi:10.1038/s41421-025-00774-4)
Supplement: Supplementary file 1 — Supplementary figures [file 41421_2025_774_MOESM1_ESM.pdf]

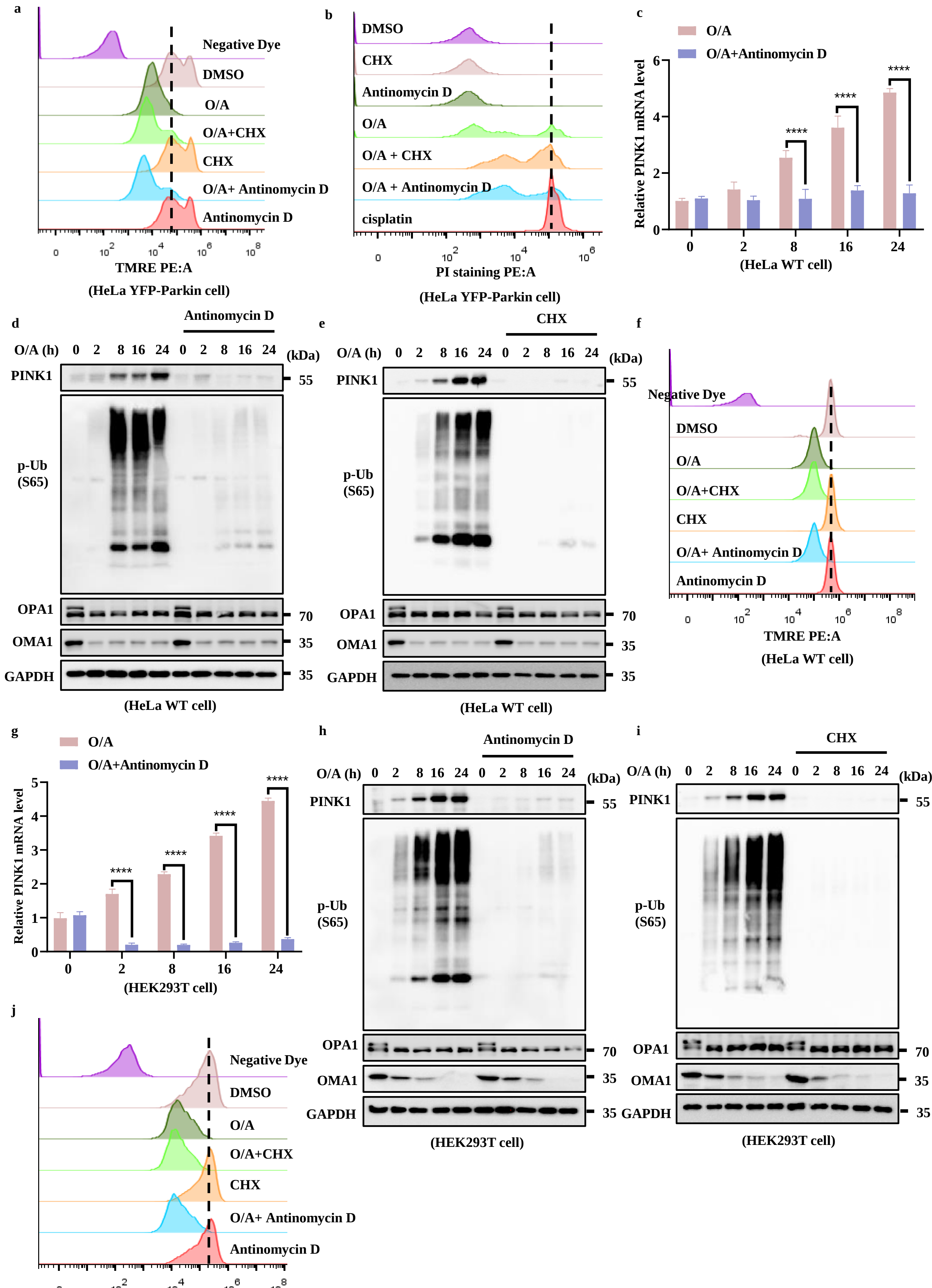
a
Negative Dye
DMSO
O/A
O/A+CHX
CHX
O/A+ Antinomycin D
Antinomycin D
TMRE PE:A
(HeLa YFP-Parkin cell)
b
DMSO
CHX
Antinomycin D
O/A
O/A + CHX
O/A + Antinomycin D
cisplatin
PI staining PE:A
(HeLa YFP-Parkin cell)
c
O/A
O/A+Antinomycin D
Relative PINK1 mRNA level
****
(HeLa WT cell)
d
Antinomycin D
O/A (h) 0 2 8 16 24 0 2 8 16 24
(kDa)
PINK1
p-Ub (S65)
OPA1
OMA1
GAPDH
55
70
35
35
(HeLa WT cell)
e
CHX
O/A (h) 0 2 8 16 24 0 2 8 16 24
(kDa)
PINK1
p-Ub (S65)
OPA1
OMA1
GAPDH
(HeLa WT cell)
f
Negative Dye
DMSO
O/A
O/A+CHX
CHX
O/A+ Antinomycin D
Antinomycin D
TMRE PE:A
(HeLa WT cell)
g
O/A
O/A+Antinomycin D
Relative PINK1 mRNA level
****
(HEK293T cell)
h
Antinomycin D
O/A (h) 0 2 8 16 24 0 2 8 16 24
(kDa)
PINK1
p-Ub (S65)
OPA1
OMA1
GAPDH
(HEK293T cell)
i
CHX
O/A (h) 0 2 8 16 24 0 2 8 16 24
(kDa)
PINK1
p-Ub (S65)
OPA1
OMA1
GAPDH
(HEK293T cell)
j
Negative Dye
DMSO
O/A
O/A+CHX
CHX
O/A+ Antinomycin D
Antinomycin D
TMRE PE:A
(HEK293T cell)

Fig S1

k

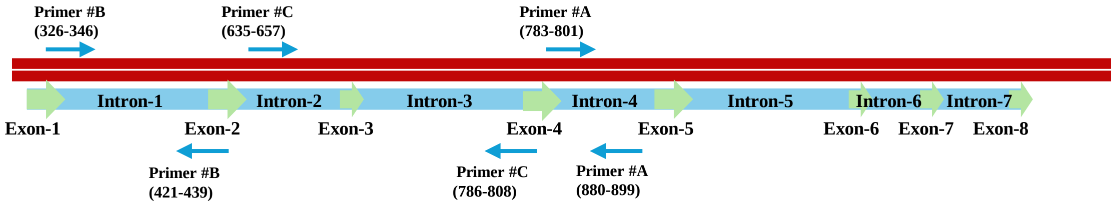

l

qPCR Primer of PINK1	Size of Product (bp)
A	117
B	114
C	174

m

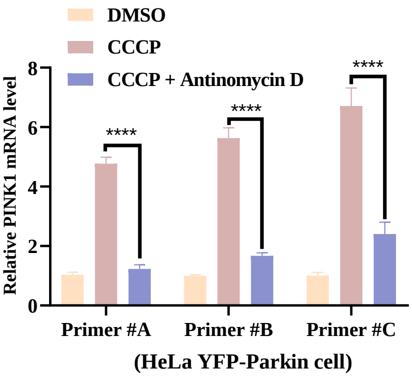

n

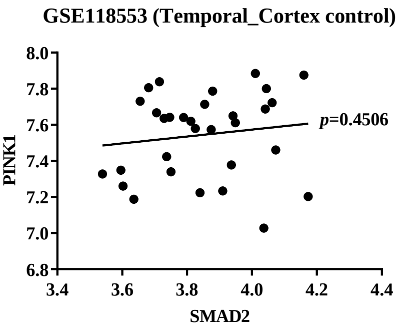

o

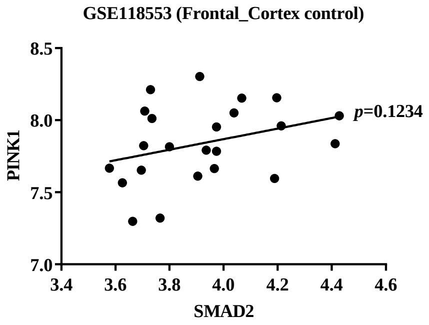

p

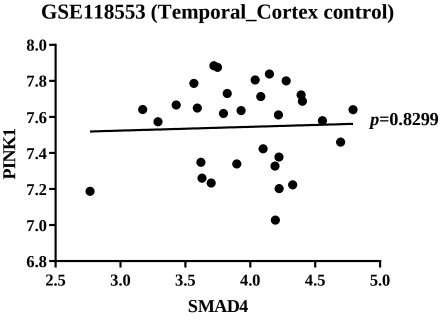

q

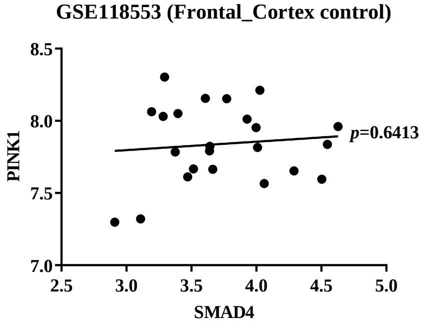

Fig S1

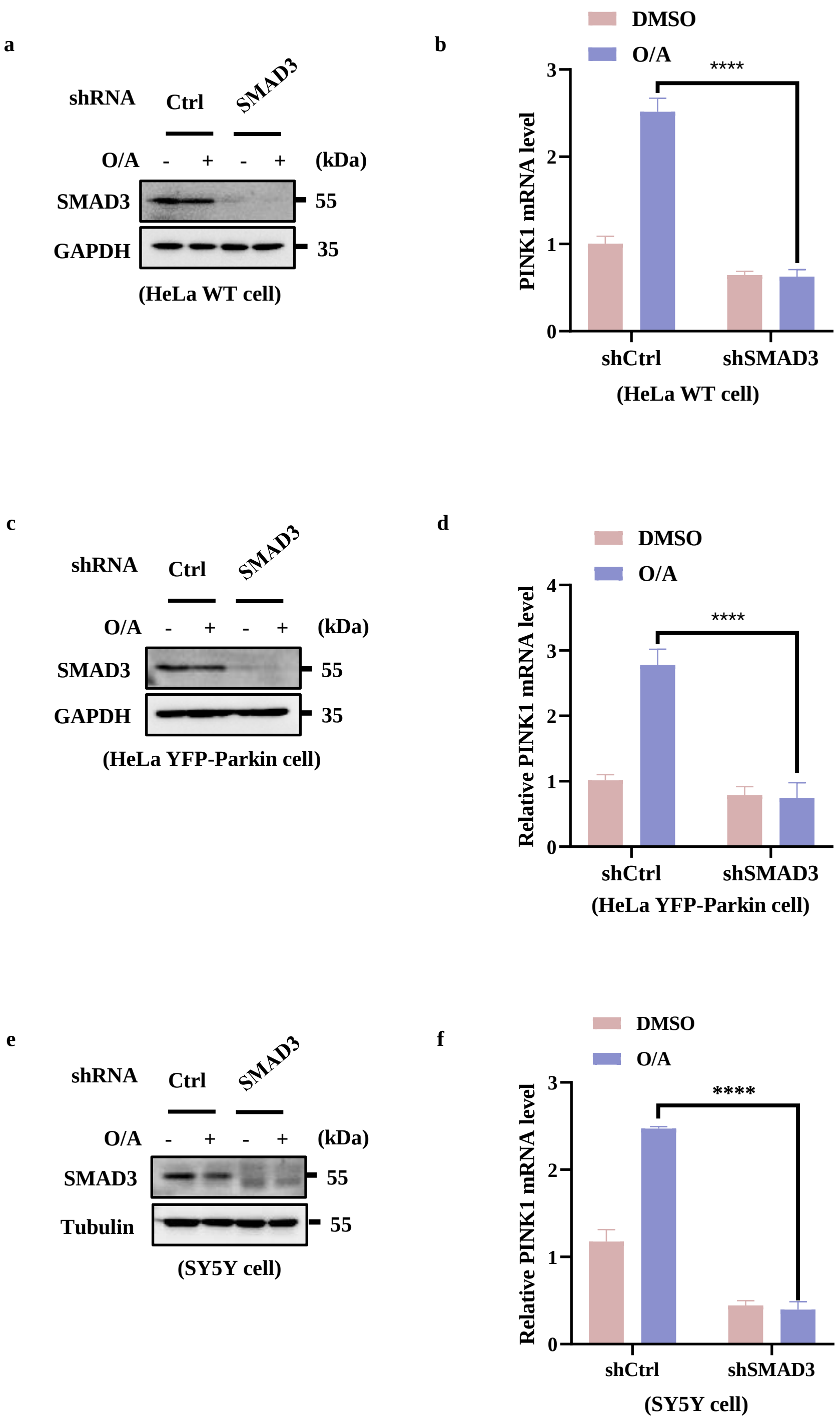
a
shRNA
Ctrl
SMAD3
O/A
- + - +
(kDa)
SMAD3
55
GAPDH
35
(HeLa WT cell)
b
DMSO
O/A
****
PINK1 mRNA level
0
1
2
3
shCtrl
shSMAD3
(HeLa WT cell)
c
shRNA
Ctrl
SMAD3
O/A
- + - +
(kDa)
SMAD3
55
GAPDH
35
(HeLa YFP-Parkin cell)
d
DMSO
O/A
****
Relative PINK1 mRNA level
0
1
2
3
4
shCtrl
shSMAD3
(HeLa YFP-Parkin cell)
e
shRNA
Ctrl
SMAD3
O/A
- + - +
(kDa)
SMAD3
55
Tubulin
55
(SY5Y cell)
f
DMSO
O/A
****
Relative PINK1 mRNA level
0
1
2
3
shCtrl
shSMAD3
(SY5Y cell)

Fig S2

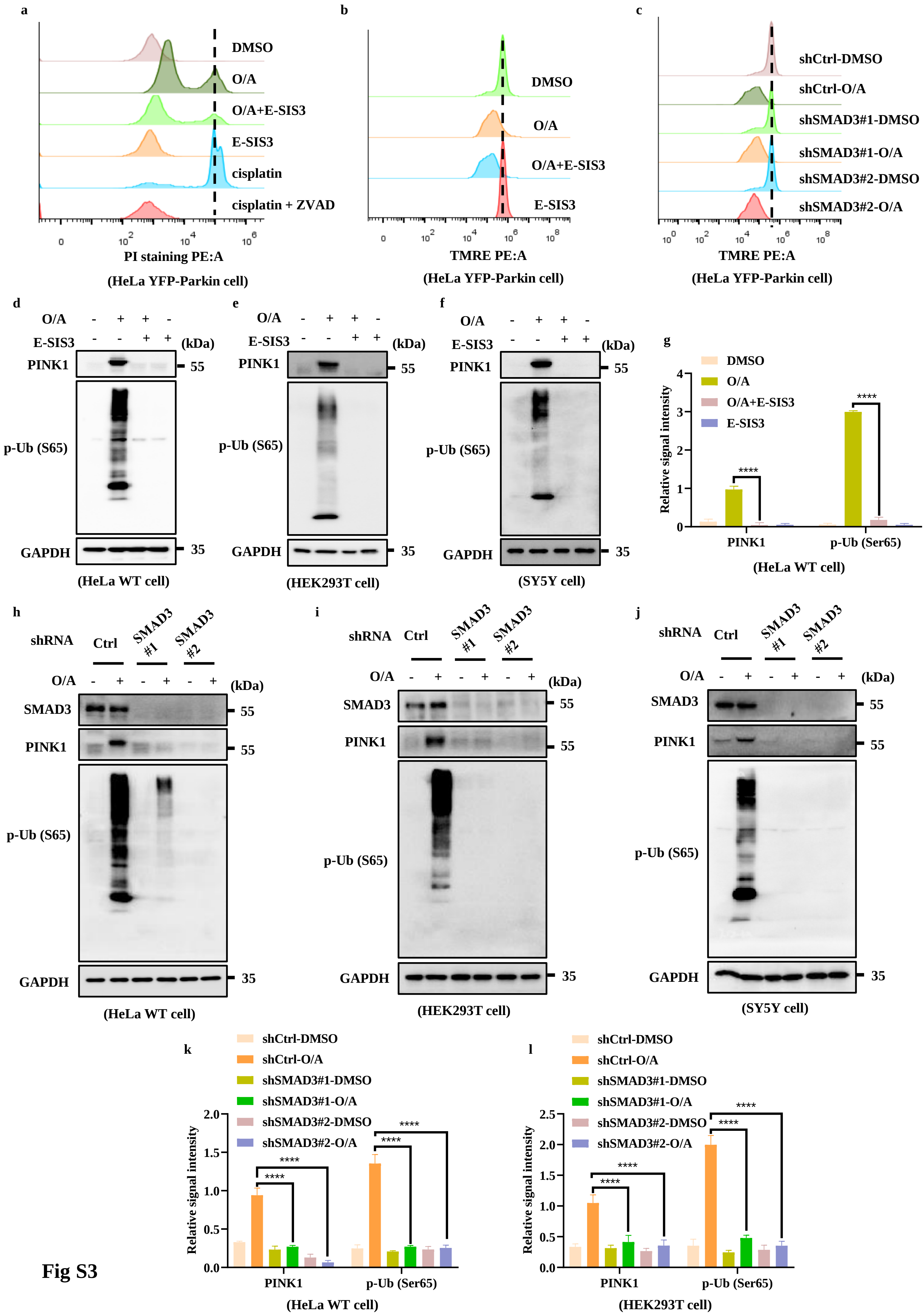
a
DMSO
O/A
O/A+E-SIS3
E-SIS3
cisplatin
cisplatin + ZVAD
PI staining PE:A
(HeLa YFP-Parkin cell)
b
DMSO
O/A
O/A+E-SIS3
E-SIS3
TMRE PE:A
(HeLa YFP-Parkin cell)
c
shCtrl-DMSO
shCtrl-O/A
shSMAD3#1-DMSO
shSMAD3#1-O/A
shSMAD3#2-DMSO
shSMAD3#2-O/A
TMRE PE:A
(HeLa YFP-Parkin cell)
d
O/A
E-SIS3
(kDa)
PINK1
p-Ub (S65)
GAPDH
55
35
(HeLa WT cell)
e
(HEK293T cell)
f
(SY5Y cell)
g
DMSO
O/A
O/A+E-SIS3
E-SIS3
Relative signal intensity
PINK1
p-Ub (Ser65)
(HeLa WT cell)
h
shRNA
Ctrl
SMAD3 #1
SMAD3 #2
SMAD3
(HeLa WT cell)
i
(HEK293T cell)
j
(SY5Y cell)
k
shCtrl-DMSO
shCtrl-O/A
shSMAD3#1-DMSO
shSMAD3#1-O/A
shSMAD3#2-DMSO
shSMAD3#2-O/A
Relative signal intensity
PINK1
p-Ub (Ser65)
(HeLa WT cell)
l
(HEK293T cell)

Fig S3

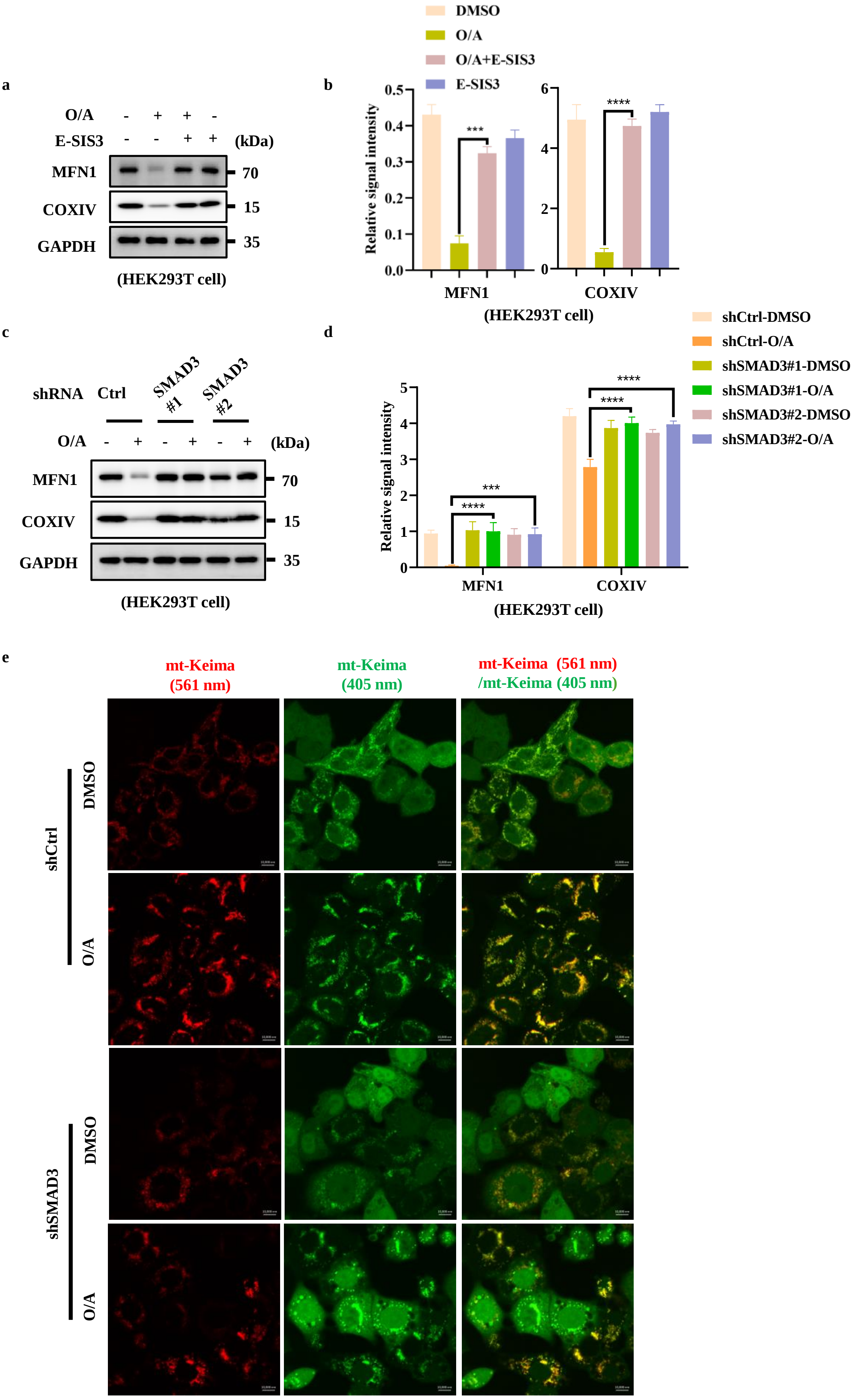
a
O/A - + + -
E-SIS3 - - + +
(kDa)
MFN1
70
COXIV
15
GAPDH
35
(HEK293T cell)
b
DMSO
O/A
O/A+E-SIS3
E-SIS3
Relative signal intensity
***
****
MFN1
COXIV
(HEK293T cell)
c
shRNA
Ctrl
SMAD3 #1
SMAD3 #2
O/A - + - + - +
(kDa)
MFN1
70
COXIV
15
GAPDH
35
(HEK293T cell)
d
shCtrl-DMSO
shCtrl-O/A
shSMAD3#1-DMSO
shSMAD3#1-O/A
shSMAD3#2-DMSO
shSMAD3#2-O/A
Relative signal intensity
***
****
MFN1
COXIV
(HEK293T cell)
e
mt-Keima (561 nm)
mt-Keima (405 nm)
mt-Keima (561 nm) /mt-Keima (405 nm)
shCtrl
DMSO
O/A
shSMAD3
DMSO
O/A

Fig S4

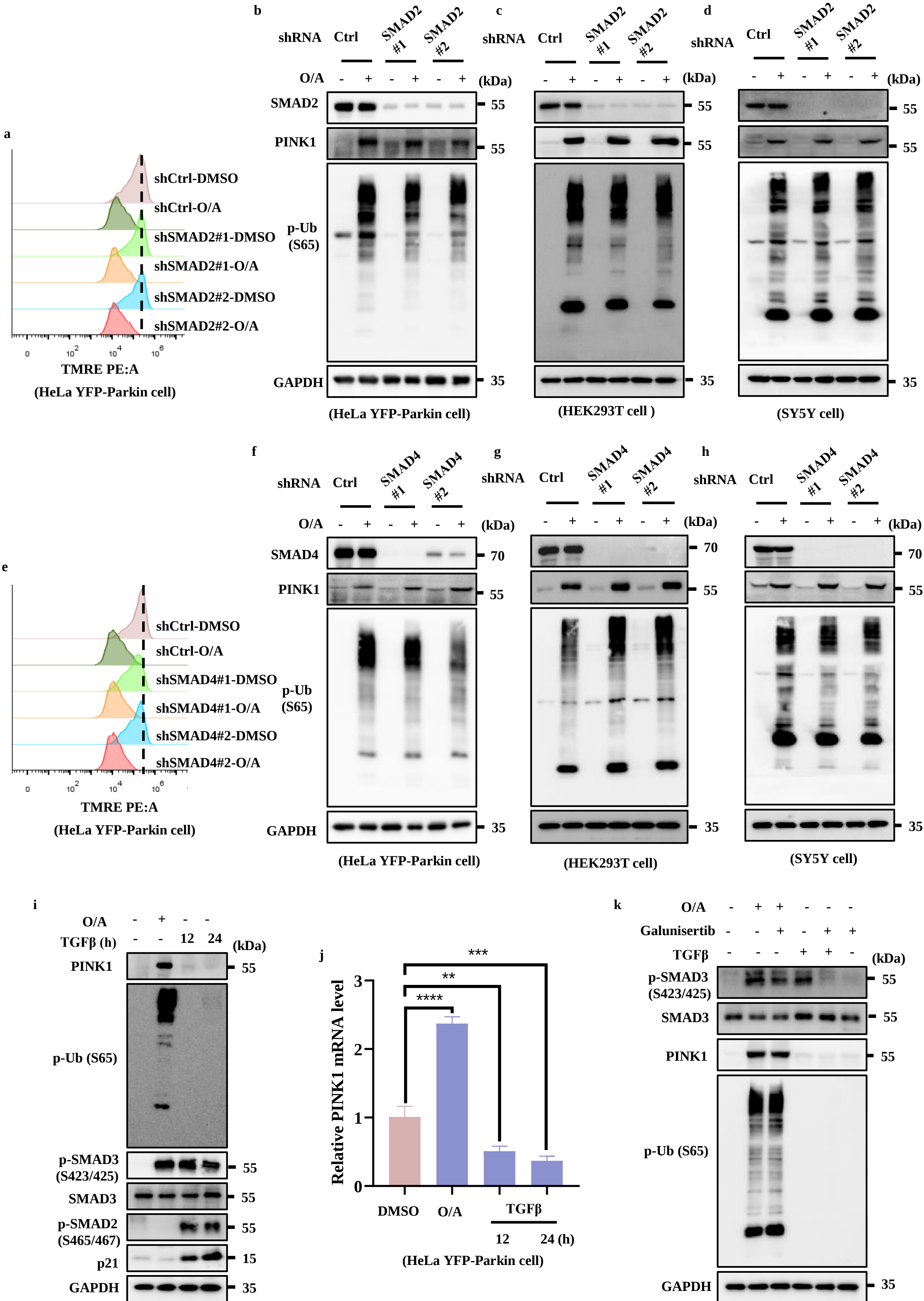

a
shCtrl-DMSO
shCtrl-O/A
shSMAD2#1-DMSO
shSMAD2#1-O/A
shSMAD2#2-DMSO
shSMAD2#2-O/A
TMRE PE:A
(HeLa YFP-Parkin cell)
b
shRNA Ctrl SMAD2 #1 SMAD2 #2
O/A - + - + - +
(kDa)
SMAD2 55
PINK1 55
p-Ub (S65)
GAPDH 35
(HeLa YFP-Parkin cell)
c
(HEK293T cell)
d
(SY5Y cell)
e
shCtrl-DMSO
shCtrl-O/A
shSMAD4#1-DMSO
shSMAD4#1-O/A
shSMAD4#2-DMSO
shSMAD4#2-O/A
TMRE PE:A
(HeLa YFP-Parkin cell)
f
shRNA Ctrl SMAD4 #1 SMAD4 #2
SMAD4 70
PINK1 55
p-Ub (S65)
GAPDH 35
(HeLa YFP-Parkin cell)
g
(HEK293T cell)
h
(SY5Y cell)
i
O/A - + - -
TGFβ (h) - - 12 24
PINK1 55
p-Ub (S65)
p-SMAD3 (S423/425) 55
SMAD3 55
p-SMAD2 (S465/467) 55
p21 15
GAPDH 35
(HeLa YFP-Parkin cell)
j
Relative PINK1 mRNA level
****
**
***
DMSO
O/A
TGFβ
12
24 (h)
(HeLa YFP-Parkin cell)
k
O/A - + + - - -
Galunisertib - - + - + +
TGFβ - - - + + -
p-SMAD3 (S423/425) 55
SMAD3 55
PINK1 55
p-Ub (S65)
GAPDH 35
(HeLa YFP-Parkin cell)

Fig S5

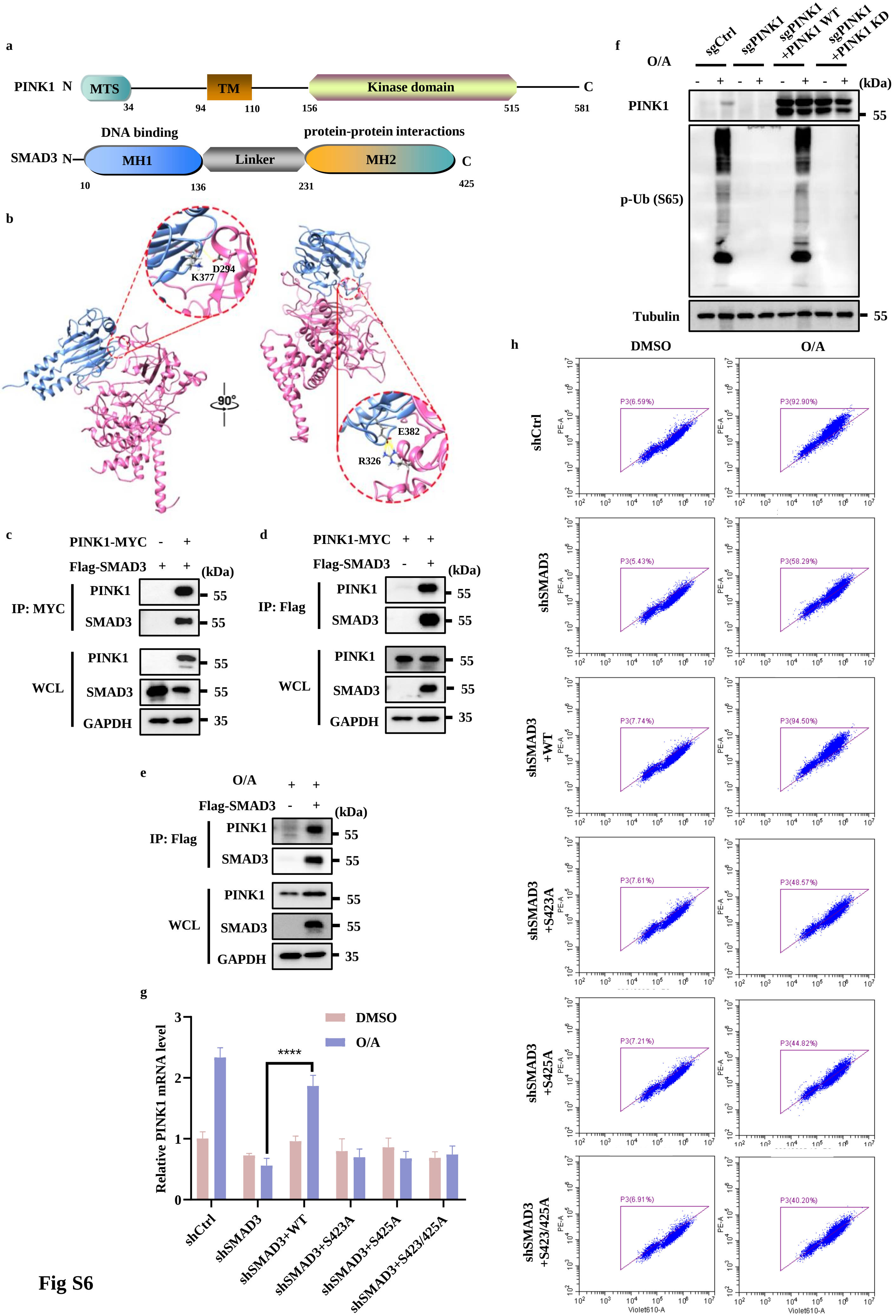

a
PINK1 N
MTS
TM
Kinase domain
C
34
94
110
156
515
581
DNA binding
protein-protein interactions
SMAD3 N
MH1
Linker
MH2
C
10
136
231
425
b
D294
K377
90°
E382
R326
c
PINK1-MYC - +
Flag-SMAD3 + +
(kDa)
IP: MYC
PINK1 55
SMAD3 55
WCL
PINK1 55
SMAD3 55
GAPDH 35
d
PINK1-MYC + +
Flag-SMAD3 - +
(kDa)
IP: Flag
PINK1 55
SMAD3 55
WCL
PINK1 55
SMAD3 55
GAPDH 35
e
O/A + +
Flag-SMAD3 - +
(kDa)
IP: Flag
PINK1 55
SMAD3 55
WCL
PINK1 55
SMAD3 55
GAPDH 35
f
O/A
sgCtrl
sgPINK1
sgPINK1 +PINK1 WT
sgPINK1 +PINK1 KD
- + - + - + - +
(kDa)
PINK1 55
p-Ub (S65)
Tubulin 55
g
Relative PINK1 mRNA level
DMSO
O/A
****
shCtrl
shSMAD3
shSMAD3+WT
shSMAD3+S423A
shSMAD3+S425A
shSMAD3+S423/425A
h
DMSO
O/A
shCtrl
P3(6.59%)
P3(92.90%)
shSMAD3
P3(5.43%)
P3(58.29%)
shSMAD3 +WT
P3(7.74%)
P3(94.50%)
shSMAD3 +S423A
P3(7.61%)
P3(48.57%)
shSMAD3 +S425A
P3(7.21%)
P3(44.82%)
shSMAD3 +S423/425A
P3(6.91%)
P3(40.20%)
PE-A
Violet610-A

Fig S6

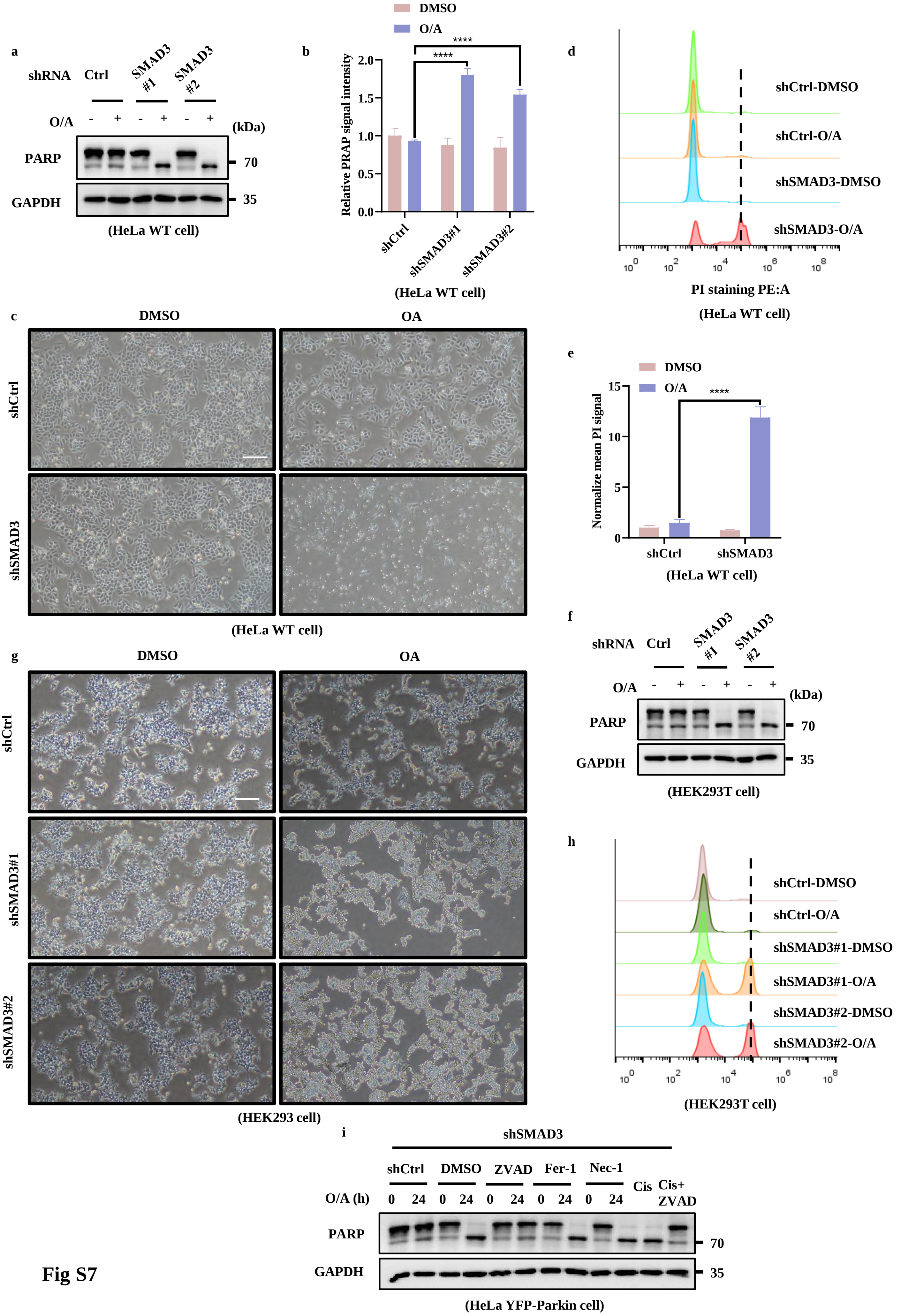

a
shRNA
Ctrl
SMAD3 #1
SMAD3 #2
O/A
(kDa)
PARP
GAPDH
70
35
(HeLa WT cell)
b
DMSO
O/A
Relative PRAP signal intensity
shCtrl
shSMAD3#1
shSMAD3#2
(HeLa WT cell)
d
shCtrl-DMSO
shCtrl-O/A
shSMAD3-DMSO
shSMAD3-O/A
PI staining PE:A
(HeLa WT cell)
c
DMSO
OA
shCtrl
shSMAD3
(HeLa WT cell)
e
DMSO
O/A
Normalize mean PI signal
shCtrl
shSMAD3
(HeLa WT cell)
f
shRNA
Ctrl
SMAD3 #1
SMAD3 #2
O/A
(kDa)
PARP
GAPDH
70
35
(HEK293T cell)
g
DMSO
OA
shCtrl
shSMAD3#1
shSMAD3#2
(HEK293 cell)
h
shCtrl-DMSO
shCtrl-O/A
shSMAD3#1-DMSO
shSMAD3#1-O/A
shSMAD3#2-DMSO
shSMAD3#2-O/A
(HEK293T cell)
i
shSMAD3
shCtrl
DMSO
ZVAD
Fer-1
Nec-1
Cis
Cis+ ZVAD
O/A (h)
PARP
GAPDH
70
35
(HeLa YFP-Parkin cell)

Fig S7

Supplementary Figure legends

Fig. S1 Association of SMAD3 with PINK1 expression.

a, f, j HeLa YFP-Parkin cells (**a**) or HeLa WT cells (**f**) or HEK293T cells (**j**) were pre-treated with with or without Antinomycin D (2 μg/mL), or CHX (50 μM) for 1 h. Subsequently, the cells were treated with or without O/A (1/1 μM) for 4 h and subjected to TRME staining for FACS analysis.

b HeLa YFP-Parkin cells pre-treated with or without Antinomycin D (2 μg/mL) or CHX (50 μM) for 24 h. Subsequently, the cells were treated with O/A (1/1 μM) for 24 h and stained with propidium iodide (PI) for FACS analysis. Cisplatin (500 μM) acted as a positive to cause sell death.

c, g PINK1 mRNA level was quantified using RT-PCR in HeLa WT cells (**c**) or HEK293T cells (**g**) followed by with or without O/A (1/1 μM) for indicated time in the presence or absence of Antinomycin D (2 μg/mL) pre-treatment.

d, e HeLa WT cells were pre-treated with or without Antinomycin D (2 μg/mL) (**d**) or CHX (50 μM) (**e**) for 1 h. Subsequently, the cells were treated with O/A (1/1 μM) for indicated time and subjected to western blotting analysis with the indicated antibodies.

h, i HEK293T cells were pre-treated with or without Antinomycin D (2 μg/mL) (**h**) or CHX (50 μM) (**i**) for 1 h. Subsequently, the cells were treated with O/A (1/1 μM) for indicated time and subjected to western blotting analysis with the indicated antibodies.

k Schematic presentation of PINK1 primers.

l The specific size of product derived from the three PINK1 primers by RT-PCR.

m PINK1 mRNA level was quantified using RT-PCR in HeLa YFP-Parkin cells followed by with or without CCCP (20 μM) for 24 h in the presence or absence of Antinomycin D (2

μg/mL) pre-treatment. *PINK1* mRNA was calculated as Relative expression of *PINK1* mRNA was calculated by $2^{-\Delta\Delta Ct}$ method and normalized to *β-actin*. Primer #A was the same as in **Fig. 1a**; and Primer #B and Primer #C were designed as two other primers for targeting different regions of PINK1 cDNA.

n, o The correlation analysis between PINK1 and SMAD2 in human temporal cortex, and frontal cortex from cohort-118553 (GEO database).

p, q The correlation analysis between PINK1 and SMAD4 in human temporal cortex, and frontal cortex from cohort-118553 (GEO database).

Data shown represent the means (±SD) of three biological replicates, $*P<0.05$, $****P<0.0001$; significance was determined by one-way ANOVA test followed by Tukey's correction (**c, g, m**).

Supplementary Fig. S2 SMAD3 is a positive nuclear transcription factor of PINK1.

a, c, e HeLa WT and SMAD3 KD (**a**), HeLa YFP-Parkin and SMAD3 KD (**c**), SY5Y and SMAD3 KD (**e**) were treated with O/A (1/1 μM) for 4 h and harvested for western blotting analysis with the indicated antibodies.

b, d, f PINK1 mRNA level was quantified using RT-PCR in HeLa WT SMAD3 KD cells (**b**), HeLa YFP-Parkin SMAD3 KD cells (**d**), SY5Y and SMAD3 KD (**f**) followed by with or without O/A (1/1 μM) for 4 h.

Data shown represent the means (±SD) of three biological replicates, $*P<0.05$, $****P<0.0001$; significance was determined by one-way ANOVA test followed by Tukey's correction (**b, d, f**).

Supplementary Fig. S3 SMAD3 up-regulates PINK1 expression.

a HeLa YFP-Parkin cells were pre-treated with E-SIS3 (20 μM) for 1 h. Subsequently, the cells were treated with or without O/A (1/1 μM) for 24 h and subjected to propidium iodide (PI) staining for FACS analysis. Cisplatin (500 μM) acted as a positive to cause sell death.

b HeLa YFP-Parkin cells were pre-treated with E-SIS3 (20 μM) for 1 h. Subsequently, the cells were treated with or without O/A (1/1 μM) for 4 h and subjected to TRME staining for FACS analysis.

c HeLa YFP-Parkin WT and SMAD3 KD were treated with O/A (1/1 μM) for 4 h and subjected to TRME staining for FACS analysis.

d, e, f HeLa WT cells (**d**) or HEK293T cells (**e**) or SY5Y cells (**f**) were pre-treated with E-SIS3 (20 μM) for 1 h. Subsequently, the cells were treated with or without O/A (1/1 μM) for 4 h and subjected to western blotting analysis with the indicated antibodies.

g The indicated proteins were quantified by normalizing to GAPDH as loading control in cells treated as in **d**.

h, i, j SMAD3 was depleted by infected with control, shSMAD3#1 or shSMAD3#2 lentiviral particles in HeLa WT cells (**h**) or HEK293T cells (**i**) or SY5Y cells (**j**). Cells were then incubated with OA (1/1 μM) for 4 h for western blotting analysis with the indicated antibodies.

k The indicated proteins were quantified by normalizing to GAPDH as loading control in cells treated as in **h**.

l The indicated proteins were quantified by normalizing to GAPDH as loading control in cells treated as in **i**.

Data shown represent the means (±SD) of three biological replicates, $*P<0.05$, $****P<0.0001$; significance was determined by one-way ANOVA test followed by Tukey's correction (**g, k, l**).

Supplementary Fig. S4 SMAD3 promotes mitophagy.

a HEK293T cells were pre-treated with E-SIS3 (20 μM) for 1 h. Subsequently, the cells were treated with or without O/A (1/1 μM) for 4 h and subjected to western blotting analysis with the indicated antibodies.

b The indicated proteins were quantified by normalizing to GAPDH as loading control in cells treated as in **a**.

c SMAD3 level was depleted by infection with control, shSMAD3#1 or shSMAD3#2 lentiviral particles in HEK293T cells. Cells were then incubated with OA (1/1 μM) for 4 h for western blotting analysis with the indicated antibodies.

d The indicated proteins were quantified by normalizing to GAPDH as loading control in cells treated as in **c**.

e Representative images of HeLa YFP-Parkin mito-Keima cells and SMAD3 KD cells treated with OA (1/1 μM) for 8 h by confocal microscopy. mt-Keima (561 nm, Red) and mt-Keima (405 nm, Green). Scale bar, 10 μm.

Data shown represent the means (±SD) of three biological replicates, **P*<0.05, *****P*<0.0001; significance was determined by one-way ANOVA test followed by Tukey's correction (**b, d**).

Supplementary Fig. S5 SMAD3 regulates PINK1 transcription independent of TGFβ signaling.

a HeLa YFP-Parkin WT and SMAD2 KD were treated with O/A (1/1 μM) for 4 h and subjected to TRME staining for FACS analysis.

b, c, d SMAD2 level was depleted by infection with control, shSMAD2 lentiviral particles in HeLa YFP-Parkin cells (**b**) or HEK293T cells (**c**) or SY5Y cells (**d**). Cells were then incubated

with OA (1/1 μM) for western blotting analysis with the indicated antibodies.

e HeLa YFP-Parkin WT and SMAD4 KD were treated with O/A (1/1 μM) for 4 h and subjected to TRME staining for FACS analysis.

f, g, h SMAD4 level was depleted by infection with control, shSMAD2 lentiviral particles in HeLa YFP-Parkin cells (**f**) or HEK293T cells (**g**) or SY5Y cells (**h**). Cells were then incubated with OA (1/1 μM) for western blotting analysis with the indicated antibodies.

i HeLa YFP-Parkin cells were treated with O/A (1/1 μM) for 4 h or TGFβ (100 ng/mL) for 12 h or 24 h and then collect the cell for western blotting analysis with the indicated antibodies.

j *PINK1* mRNA level was quantified using RT-PCR in HeLa YFP-Parkin cells followed by O/A (1/1 μM) for 4 h or TGFβ (100 ng/mL) for 12 h or 24 h.

k HeLa YFP-Parkin cells were pretreated with Galunisertib (20 μM) for 1 h. Subsequently, the cells were treated with or without O/A (1/1 μM) or TGFβ (100 ng/mL) for 4 h and subjected to western blotting analysis with the indicated antibodies.

Data shown represent the means (±SD) of three biological replicates, **P*<0.05, *****P*<0.0001; significance was determined by one-way ANOVA test followed by Tukey's correction (**j**).

Supplementary Fig. S6 SMAD3 is phosphorylated by PINK1.

a The functional domains of PINK1 and SMAD3 proteins.

b The MD simulations for the interaction site between PINK1 and SMAD3 based on the Alphafold2 model.

c Lysates of HeLa YFP-Parkin cells overexpressing Flag-SMAD3 and/or PINK1-MYC were subjected to reciprocal co-immunoprecipitation (co-IP) to detect protein interaction.

d Lysates of HeLa YFP-Parkin cells overexpressing PINK1-MYC and/or Flag-SMAD3 were subjected to reciprocal co-immunoprecipitation (co-IP) to detect protein interaction.

e Lysates of HeLa YFP-Parkin cells were treated with/without O/A (1/1 μM) for 4 h followed by overexpressing Flag-SMAD3, and then subjected to reciprocal co-immunoprecipitation (co-IP) to detect protein interaction.

f PINK1 was depleted by infected with control or sgPINK1 lentiviral particles in HeLa WT cells. Cells were then incubated with OA (1/1 μM) followed by PINK1-WT-V5/PINK1-KD-V5 overexpression for western blotting analysis with the indicated antibodies.

g *PINK1* mRNA level was quantified by RT-PCR in HeLa YFP-Parkin WT and SMAD3 KD cells after transfected with Flag-SMAD3, Flag-SMAD3 S423A, Flag-SMAD3 S425A or Flag-SMAD3 S423/425A for 24 hours with/without O/A (1/1 μM) for 4 h.

h HeLa YFP-Parkin Keima WT and SMAD3 KD cells transfected with Flag-SMAD3, Flag-SMAD3 S423A, Flag-SMAD3 S425A or Flag-SMAD3 S423/425A for 24 hours were treated with O/A (1/1 μM) for 8 h and harvested for FACS analysis by excitation at 405 nm (neutral pH) and 561 nm (acidic pH).

Data shown represent the means (±SD) of three biological replicates, *P<0.05, ****P< 0.0001; significance was determined by one-way ANOVA test followed by Tukey's correction (**g**).

Supplementary Fig. S7 Activation of SMAD3 provides a pro-survival mechanism against mitochondrial stress.

a HeLa WT and SMAD3 KD cells were treated with either DMSO or O/A (1 μM) for 24 h. The cells were then harvested for western blotting analysis with the indicated antibodies.

b The indicated proteins were quantified by normalizing to GAPDH as loading control in cells treated as in **a**.

c HeLa WT and SMAD3 KD cells were treated with either DMSO or O/A (1 μM) for 24 h. The cells were then imaged with phase contrast microscopy. Scale bar: 50 μm.

d HeLa WT and SMAD3 KD cells treated as in Supplementary Fig. **S7c** were stained with propidium iodide (PI) and subjected to FACS analysis.

e The relative mean PE signaling was quantified by normalizing to DMSO group in cells treated as in **d**.

f HEK293T and SMAD3 KD cells were treated with either DMSO or O/A (1 μM) for 24 h. The cells were then harvested for western blotting analysis with the indicated antibodies.

g HEK293T and SMAD3 KD cells were treated with either DMSO or O/A (1 μM) for 24 h. The cells were then imaged with phase contrast microscopy. Scale bar: 50 μm.

h HEK293T and SMAD3 KD cells treated as in Supplementary Fig. **S7g** were stained with propidium iodide (PI) and subjected to FACS analysis.

i HeLa WT and SMAD3 KD cells were pre-treated with Z-VAD-FMK (20 μM) or Ferrostatin-1 (2 μM) or Necrostatin-1 (30 μM) and then treated with either DMSO or O/A (1 μM) for 24 h. The cells were then harvested for western blotting analysis with the indicated antibodies. Cisplatin (500 μM) acted as a positive to cause sell death.

Data shown represent the means (±SD) of three biological replicates, **P*<0.05, *****P*<0.0001; significance was determined by one-way ANOVA test followed by Tukey's correction (**b, e**).
